# Dimensionless Groups by Entropic Similarity: II—Wave Phenomena and Information-Theoretic Flow Regimes

**DOI:** 10.3390/e25111538

**Published:** 2023-11-11

**Authors:** Robert K. Niven

**Affiliations:** School of Engineering and Technology, The University of New South Wales, Canberra, ACT 2600, Australia; r.niven@adfa.edu.au

**Keywords:** dimensional analysis, entropic similarity, compressible flow, open channel flow, gravity flow, electromagnetic radiation

## Abstract

The aim of this study is to explore the insights of the information-theoretic definition of similarity for a multitude of flow systems with wave propagation. This provides dimensionless groups of the form $\Pi_{\mathrm{info}}=U/c$, where *U* is a characteristic flow velocity and *c* is a signal velocity or wave celerity, to distinguish different information-theoretic flow regimes. Traditionally, dimensionless groups in science and engineering are defined by *geometric similarity,* based on ratios of length scales; *kinematic similarity,* based on ratios of velocities or accelerations; and *dynamic similarity,* based on ratios of forces. In Part I, an additional category of *entropic similarity* was proposed based on ratios of (i) entropy production terms; (ii) entropy flow rates or fluxes; or (iii) information flow rates or fluxes. In this Part II, the information-theoretic definition is applied to a number of flow systems with wave phenomena, including acoustic waves, blast waves, pressure waves, surface or internal gravity waves, capillary waves, inertial waves and electromagnetic waves. These are used to define the appropriate Mach, Euler, Froude, Rossby or other dimensionless number(s)—including new groups for internal gravity, inertial and electromagnetic waves—to classify their flow regimes. For flows with wave dispersion, the coexistence of different celerities for individual waves and wave groups—each with a distinct information-theoretic group—is shown to imply the existence of more than two information-theoretic flow regimes, including for some acoustic wave systems (subsonic/mesosonic/supersonic flow) and most systems with gravity, capillary or inertial waves (subcritical/mesocritical/supercritical flow). For electromagnetic wave systems, the additional vacuum celerity implies the existence of four regimes (subluminal/mesoluminal/transluminal/superluminal flow). In addition, entropic analyses are shown to provide a more complete understanding of frictional behavior and sharp transitions in compressible and open channel flows, as well as the transport of entropy by electromagnetic radiation. The analyses significantly extend the applications of entropic similarity for the analysis of flow systems with wave propagation.

## 1. Introduction

Since the seminal work of Buckingham [1], built on the insights of many predecessors [2,3,4,5,6,7,8,9], dimensional analysis and similarity arguments based on dimensionless groups have provided a powerful tool—and in many cases, the most important tool—for the analysis of physical, chemical, biological, geological, environmental, astronomical, mechanical and thermodynamic systems, especially those involving fluid flow. The dimensionless groups obtained are usually classified into those arising from *geometric similarity*, based on ratios of length scales (or areas or volumes); *kinematic similarity*, based on ratios of velocities or accelerations; and *dynamic similarity*, based on ratios of forces [10,11,12,13,14,15,16,17]. Thus, for example, the Froude number [18] is often interpreted by dynamic similarity as [10,12,19]:

(1) $$\begin{array}{c} Fr=\frac{\mathrm{inertial}\;\mathrm{force}}{\mathrm{gravity}\;\mathrm{force}}=\frac{F_{I}}{F_{g}}∼\frac{\rho \ell^{3}\left(U^{2}/\ell \right)}{\rho g\ell^{3}}=\frac{U^{2}}{g\ell } \end{array}$$

(or as the square root), where ∼ indicates “of the order of” (discarding numerical constants), ρ is the fluid density [SI units: kg m${}^{-3}$], *ℓ* is an applicable length scale [m], *U* is a velocity scale [m s${}^{-1}$] and *g* is the acceleration due to gravity [m s${}^{-2}$]. In common with many dimensionless groups, Equation (1) provides an identifier of the flow regime, in this case the dominance of gravity forces at low Froude numbers, associated with subcritical flow; and the dominance of inertial forces at high Froude numbers, producing supercritical flow.

Part I of this study [20] proposes a new interpretation for a large class of dimensionless groups based on the principle of *entropic similarity*, involving ratios of (i) entropy production terms; (ii) entropy flow rates or fluxes; or (iii) information flow rates or fluxes. Since all processes involving work against friction, dissipation, diffusion, dispersion, mixing, separation, chemical reaction, gain of information or other irreversible changes are driven by (or must overcome) the second law of thermodynamics, it is appropriate to analyze these processes directly in terms of competing entropy-producing and transporting phenomena and the dominant entropic regime, rather than indirectly in terms of forces. The entropic perspective is shown to provide a new entropic interpretation of many known dimensionless groups, as well as a number of new groups [20], significantly expanding the scope of dimensional arguments for the resolution of new and existing problems.

The aim of this Part II study is to explore the insights of the information-theoretic definition of similarity, by application to a multitude of fluid flow systems subject to various wave phenomena. This has application to a wide range of fluid flow systems, including compressible flows (acoustic waves), enclosed flows (pressure waves), open channel flows, lakes and oceans (surface gravity and capillary waves), oceanographic and meteorological flows (internal gravity waves and internal waves), and subatomic particle flows (electromagnetic waves). This work is set out as follows. In Section 3, we provide a recap of entropy concepts, dimensionless groups and the principle of entropic similarity, including the information-theoretic definition. In Section 4, we then examine a number of flow systems with wave phenomena. For each wave type, the information-theoretic definition of similarity and its role as a discriminator between different information-theoretic flow regimes is examined in detail, leading to a revised interpretation of several known dimensionless groups and a number of new groups. For systems with wave dispersion, the analyses also suggest the possibility of more than two information-theoretic flow regimes. For several flow types, the information-theoretic flow regimes, combined with a direct entropic analysis (applying the second law of thermodynamics), are also shown to provide a more complete understanding of the observed frictional flow regimes and the occurrence of sharp transitions. The findings are summarized in the conclusions in Section 5.

## 2. Theoretical Foundations

The (dimensionless) discrete entropy and relative entropy functions are [21,22,23,24,25,26]:

(2) $$H_{\mathrm{Sh}}=-\sum_{i=1}^{n}p_{i}\ln p_{i}\;\mathrm{and}\;H=-\sum_{i=1}^{n}p_{i}\ln\frac{p_{i}}{q_{i}}$$

where $p_{i}$ is the probability of the *i*th outcome, from *n* such outcomes, and $q_{i}$ is the prior probability of the *i*th outcome. The second form reduces to the first, plus a constant, in the case of equal prior probabilities. Both functions and their continuous form can be derived from the axioms of a measure of uncertainty [21,26,27] or from the combinatorial definition of entropy [28,29].

The dimensionless entropy concept (2) provides the foundation for the thermodynamic entropy $S=k_{B}H^{*}$ [J K${}^{-1}$], where $k_{B}$ is the Boltzmann constant [J K${}^{-1}$] and $H^{*}$ is the maximum entropy [23,24,25,30,31]. By analysis of the thermodynamic entropy balance in an open system, it is possible to derive expressions for the global and local entropy production, respectively, within an integral control volume or an infinitesimal fluid element [32,33,34,35,36,37]:

(3) $$\begin{array}{c} \dot{\sigma }=\underset{CV}{\;\int \int \int \;}\hat{\dot{\sigma }}dV=\underset{CV}{\;\int \int \int \;}\left[\frac{\partial }{\partial t}\rho s+\nabla \cdot \left(j_{S}+\rho su\right)\right]dV\geq 0 \end{array}$$

(4) $$\begin{array}{c} \hat{\dot{\sigma }}=\frac{\partial }{\partial t}\rho s+\nabla \cdot \left(j_{S}+\rho su\right)\geq 0 \end{array}$$

where CV is the control volume, *t* is time [s], *s* is the specific entropy (per unit mass of fluid) [J K${}^{-1}$ kg${}^{-1}$], $j_{S}$ is the non-fluid entropy flux [J K${}^{-1}$ m${}^{-2}$ s${}^{-1}$], u is the fluid velocity [m s${}^{-1}$], dV is a volume element [m${}^{3}$], n is an outwardly directed unit normal [-] and ∇ is the Cartesian nabla operator [m${}^{-1}$]. The inequalities in Equations (3) and (4) express the global and local definitions of the second law of thermodynamics, applicable to an open system.

In information theory, it is usual to rewrite Equation (2) as the binary entropy and relative entropy, expressed in binary digits or “bits” [38]:

(5) $$\begin{array}{c} B_{\mathrm{Sh}}=-\sum_{i=1}^{n}p_{i}\log_{2}p_{i}\;\mathrm{and}\;B=-\sum_{i=1}^{n}p_{i}\log_{2}\frac{p_{i}}{q_{i}} \end{array}$$

Comparing Equation (2), we obtain $H_{\mathrm{Sh}}=B_{\mathrm{Sh}}\ln2$ and H=Bln2. The change in information about a system can then be defined as the negative change in its binary entropy [39,40,41,42,43,44]:

(6) $$\Delta I=-\Delta B_{\mathrm{Sh}}\;\mathrm{or}\;\Delta I=-\Delta B$$

For example, consider a coin toss with equiprobable outcomes H and T. Without knowledge of the outcome, an observer will assign $p_{H}=p_{T}=\frac{1}{2}$; hence, the entropy in Equation (5) gives $B_{\mathrm{Sh}}^{\mathrm{init}}=1$ bit. Once informed of the outcome, the observer must assign one probability to zero and the other to unity, hence $B_{\mathrm{Sh}}^{\mathrm{final}}=0$ bits and $\Delta B_{\mathrm{Sh}}=0-1=-1$ bit. From Equation (6), the information gained in the binary decision is $\Delta I=-\Delta B_{\mathrm{Sh}}=1$ bit [21,38,45]. If instead we use the relative entropy in Equation (5) with equal priors $q_{H}=q_{T}=\frac{1}{2}$, we obtain $B^{\mathrm{init}}=0$ bits, $B^{\mathrm{final}}=-1$ bits and ΔB=−1−0=−1 bits, so again ΔI=1 bit.

This idea was taken further by Szilard [45] and later authors based on analyses of Maxwell’s demon [40,41,42,43,46,47], to establish a fundamental relationship between changes in information, thermodynamic entropy and energy. From the second law of thermodynamics:

(7) $$\begin{array}{c} \Delta S_{\mathrm{univ}}=\Delta S_{\mathrm{sys}}+\Delta S_{\mathrm{ROU}}\geq 0 \end{array}$$

where $\Delta S_{\mathrm{univ}}$ is the change in thermodynamic entropy of the universe [J K${}^{-1}$], which can be partitioned into the changes $\Delta S_{\mathrm{sys}}$ within a system and $\Delta S_{\mathrm{ROU}}$ in the rest of the universe [48]. Substituting for $\Delta S_{\mathrm{sys}}$ using Equations (5) and (6) gives:

(8) $$\begin{array}{c} k_{B}\ln2\;\Delta B+\Delta S_{\mathrm{ROU}}=-k_{B}\ln2\;\Delta I+\Delta S_{\mathrm{ROU}}\geq 0 \end{array}$$

In general, a system can only influence the entropy of the rest of the universe by a transfer of disordered energy $\Delta E=T\Delta S_{\mathrm{ROU}}$ [J], where *T* is the absolute temperature [K], such as that carried by heat or chemical species. Rearranging Equation (8) and substituting for ΔE gives:

(9) $$\begin{array}{c} k_{B}\ln2\;\Delta I\leq \Delta S_{\mathrm{ROU}}\;\mathrm{or}\;k_{B}T\ln2\;\Delta I\leq \Delta E \end{array}$$

or in time rate form:

(10) $$\begin{array}{c} k_{B}\ln2\;\frac{\partial I}{\partial t}\leq \frac{\partial S_{\mathrm{ROU}}}{\partial t}\;\mathrm{or}\;k_{B}T\ln2\;\frac{\partial I}{\partial t}\leq \frac{\partial E}{\partial t} \end{array}$$

Equations (9) and (10) provide an information-theoretic formulation of the second law of thermodynamics, in which each bit of information gained by an observer about a system must be paid for by an energy cost of at least $k_{B}T\ln2$, or an entropy cost of at least $k_{B}\ln2$. This imposes a fundamental limit on processes that involve the transmission of information: if the penalty incurred in Equations (9) and (10) is not paid, then the information will not be transmitted.

## 3. Dimensionless Groups and the Principle of Entropic Similarity

As discussed in Part I [20], a *dimensionless group* is a unitless parameter used to represent an attribute of a physical system, independent of the system of units used. These can be identified and applied in three ways. First, the matching of dimensionless groups between a system (prototype) and its model, known as *similarity* or *similitude*, can be used for experimental scaling [5,7]. Second, the governing dimensionless groups for a system can be extracted from its list of parameters by the method of *dimensional analysis* [1]. Third, the governing partial differential equation(s) for a system can be converted into dimensionless form, known as *non-dimensionalization*, to identify its governing dimensionless groups [11,12,14,15,16,49]. Recently, it was shown that the one-parameter Lie group of point transformations provides a rigorous method for the non-dimensionalization of a differential equation, based on the intrinsic dimensions of the system [50]. For over a century, dimensional methods have been recognized as powerful tools—and in many cases, the primary tools—for the analysis of a wide range of systems across all branches of science and engineering [51,52,53,54,55,56,57].

Dimensionless groups—commonly labeled Π—can be classified as [10,11,12,13,14,15,16,17]:

(i) Those arising from *geometric similarity*, based on ratios of length scales $\ell_{i}$ [m] or associated areas or volumes:
  (11) $$\begin{array}{c} \Pi_{\mathrm{geom}}=\frac{\ell_{1}}{\ell_{2}}\;\;\mathrm{or}\;\;\Pi_{\mathrm{geom}}=\frac{\ell_{1}^{2}}{\ell_{2}^{2}}\;\;\mathrm{or}\;\;\Pi_{\mathrm{geom}}=\frac{\ell_{1}^{3}}{\ell_{2}^{3}} \end{array}$$
(ii) Those arising from *kinematic similarity*, based on ratios of magnitudes of velocities $U_{i}$ [m s${}^{-1}$] or accelerations $a_{i}$ [m s${}^{-2}$]:
  (12) $$\begin{array}{c} \Pi_{\mathrm{kinem}}=\frac{U_{1}}{U_{2}}\;\;\mathrm{or}\;\;\Pi_{\mathrm{kinem}}=\frac{a_{1}}{a_{2}} \end{array}$$
(iii) Those arising from *dynamic similarity*, based on ratios of magnitudes of forces $F_{i}$ [N]:
  (13) $$\begin{array}{c} \Pi_{\mathrm{dynam}}=\frac{F_{1}}{F_{2}} \end{array}$$

In Part I [20], an additional category of dimensionless groups was proposed based on *entropic similarity*, based on the following definitions:

(i) Those defined by ratios of global or local entropy production terms:
  (14) $$\begin{array}{c} \Pi_{\mathrm{entrop}}=\frac{{\dot{\sigma }}_{1}}{{\dot{\sigma }}_{2}}\;\;\mathrm{or}\;\;{\hat{\Pi }}_{\mathrm{entrop}}=\frac{{\hat{\dot{\sigma }}}_{1}}{{\hat{\dot{\sigma }}}_{2}} \end{array}$$
  where Π represents a global or summary dimensionless group, $\hat{\Pi }$ is a local group, ${\dot{\sigma }}_{i}$ is the global entropy production by the *i*th process (3) [J K${}^{-1}$ s${}^{-1}$] and ${\hat{\dot{\sigma }}}_{i}$ is the local entropy production by the *i*th process (4) [J K${}^{-1}$ m${}^{-3}$ s${}^{-1}$].
(ii) Those defined by ratios of global flow rates of thermodynamic entropy, or by components or magnitudes of their local fluxes:
  (15) $$\begin{array}{c} \Pi_{\mathrm{entrop}}=\frac{F_{S,1}}{F_{S,2}}\;\;\mathrm{or}\;\;{\hat{\Pi }}_{\mathrm{entrop}}\left(n\right)=\frac{j_{S_{1}}\cdot n}{j_{S_{2}}\cdot n}\;\;\mathrm{or}\;\;{\hat{\Pi }}_{\mathrm{entrop}}=\frac{\left|\right|j_{S_{1}}\left|\right|}{\left|\right|j_{S_{2}}\left|\right|} \end{array}$$
  where $F_{S,i}$ is the entropy flow rate of the *i*th process [J K${}^{-1}$ s${}^{-1}$], $j_{S_{i}}$ is the non-fluid entropy flux of the *i*th process [J K${}^{-1}$ m${}^{-2}$ s${}^{-1}$] (see (4)), n is a unit normal and $\left||a|\right|=\sqrt{a^{⊤}a}$ is the Euclidean norm for vector a.
(iii) Those defined by an information-theoretic threshold, for example by the ratio of the local information flux carried by the flow $j_{I,\mathrm{flow}}$ [bits m${}^{-2}$ s${}^{-1}$] to that transmitted by a carrier of information $j_{I,\mathrm{signal}}$ [bits m${}^{-2}$ s${}^{-1}$]:
  (16) $$\begin{array}{c} {\hat{\Pi }}_{\mathrm{info}}=\frac{\left|\right|j_{I,\mathrm{flow}}\left|\right|}{\left|\right|j_{I,\mathrm{signal}}\left|\right|}=\frac{\left|\right|\rho_{I,\mathrm{flow}}\;u_{\mathrm{flow}}\left|\right|}{\left|\right|\rho_{I,\mathrm{signal}}\;u_{\mathrm{signal}}\left|\right|} \end{array}$$
  In this perspective, flows in which the information flux of the fluid exceeds that of a signal (${\hat{\Pi }}_{\mathrm{info}}>1$) will experience a different information-theoretic flow regime to those in which the signal flux dominates (${\hat{\Pi }}_{\mathrm{info}}<1$). In Equation (16), each information flux is further reduced to the product of an information density $\rho_{I}$ [bits m${}^{-3}$] and the corresponding fluid velocity $u_{\mathrm{flow}}$ or signal velocity $u_{\mathrm{signal}}$ [m s${}^{-1}$]. Making the strong assumption that the two information densities are comparable, Equation (16) simplifies to give the local or summary kinematic definitions:
  (17) $$\begin{array}{c} {\hat{\Pi }}_{\mathrm{info}}=\frac{\left|\right|u_{\mathrm{flow}}\left|\right|}{\left|\right|u_{\mathrm{signal}}\left|\right|},\;\Pi_{\mathrm{info}}=\frac{U_{\mathrm{flow}}}{U_{\mathrm{signal}}} \end{array}$$
  where $U_{\mathrm{flow}}$ and $U_{\mathrm{signal}}$ are representative flow and signal velocities [m s${}^{-1}$].

In Part I [20], the first two definitions of entropic similarity in Equations (14) and (15) were applied to a range of diffusion, chemical reaction and dispersion phenomena, to reveal the entropic interpretation of many known dimensionless groups, and to define a number of new groups. To continue the application of entropic similarity to flow processes, it is necessary to examine the third definition based on information-theoretic similarity in Equation (17), and its application to flows with wave motion.

## 4. Wave Motion and Information-Theoretic Flow Regimes

A *wave* can be defined as an oscillatory process that facilitates the transfer of energy through a medium or free space. Generally, this is governed by the wave equation:

(18) $$\begin{array}{cc} \frac{\partial^{2}\phi }{\partial t^{2}} & =c^{2}\nabla^{2}\phi \end{array}$$

where ϕ is a displacement parameter and *c* is the characteristic wave velocity (*celerity*) [m s${}^{-1}$], measured relative to the medium. Wave motion, in its own right, does not produce entropy, although some waves are carriers of entropy, and wave interactions with materials or boundaries can be dissipative in some situations. However, a wave is also a *carrier of information*, communicating the existence and strength of a disturbance or source of energy. Its celerity therefore provides an intrinsic velocity scale for the rate of transport of information through the medium. For a fluid flow with local velocity u, the celerity provides a threshold between two different information-theoretic flow regimes, which respectively can (||u||<c) or cannot (||u||>c) be influenced by downstream disturbances. Adopting the information-theoretic formulation of similarity (17) and allowing for a vector celerity field c with magnitude ||c||=c, this can be used to define the local directional, local vector, macroscopic vector and macroscopic scalar dimensionless groups, respectively:

(19) $$\begin{array}{c} {\hat{\Pi }}_{\mathrm{info}}\left(n\right)=\frac{u\cdot n}{c\cdot n},\;{\hat{\Pi }}_{\mathrm{info}}=\frac{u}{c},\;\Pi_{\mathrm{info}}=\frac{U}{c},\;\Pi_{\mathrm{info}}=\frac{U}{c} \end{array}$$

where n is a given unit normal. The first group provides a local direction-dependent definition based on a vector celerity, but will exhibit a singularity as n→c. To overcome this, an alternative definition can be adopted based on component-wise division [20]:

(20) $$\begin{array}{c} {\tilde{\Pi }}_{\mathrm{info}}=u⊘c \end{array}$$

where ⊘ is the component-wise (Hadamard) division operator. The second definition in Equation (19) gives a local vector definition with a scalar celerity, the third gives a vector definition based on a summary velocity vector U [m s${}^{-1}$], and the last gives a summary criterion based on a summary velocity magnitude *U* [m s${}^{-1}$]. Each of these groups provides a discriminator between two information-theoretic flow regimes separated by the critical value of 1 (or for some definitions, the vector 1), governed respectively by downstream-controlled or upstream-controlled processes.

In the following sections, we examine several wave types from this perspective. For some flows, a sharp junction can be formed between the two information-theoretic flow regimes (e.g., a shock wave or hydraulic jump), with a high rate of entropy production. Wave-carrying flows are also subject to friction, with distinct differences between the two flow regimes. Both these features are explored for several flows, drawing on all definitions of entropic similarity in Equations (14)–(17) as needed. Due to common practice, this study makes some excursions from the notation used in Part I [20]; these are mentioned explicitly.

### 4.1. Acoustic Waves

#### 4.1.1. Mach Numbers and Compressible Flow Regimes

An acoustic or sound wave carries energy through a material by longitudinal compression and decompression at the sonic velocity $a=\sqrt{dp/d\rho }=\sqrt{K/\rho }$ [m s${}^{-1}$], where *p* is the pressure [Pa] and *K* is the bulk modulus of elasticity [Pa] [10,11,12,58,59,60]. For isentropic (adiabatic and reversible) changes in an ideal gas, this reduces to $a=\sqrt{\gamma p/\rho }=\sqrt{\gamma R^{*}T}$, where γ is the adiabatic index [-] and $R^{*}$ is the specific gas constant [J K${}^{-1}$ kg${}^{-1}$]. By information-theoretic similarity (19), this can be used to define the local scalar and macroscopic Mach numbers:

(21) $$\begin{array}{c} {\hat{\Pi }}_{a}=\hat{M}=\frac{\left||u|\right|}{a}\overset{\mathrm{ideal}\;\mathrm{gas}}{\underset{\mathrm{isentropic}}{\to }}\frac{\left||u|\right|}{\sqrt{\gamma R^{*}T}},\;\Pi_{a}=M_{\infty }=\frac{U_{\infty }}{a_{\infty }}\overset{\mathrm{ideal}\;\mathrm{gas}}{\underset{\mathrm{isentropic}}{\to }}\frac{U_{\infty }}{\sqrt{\gamma R^{*}T_{\infty }}} \end{array}$$

where $U_{\infty }$ is the free-stream fluid velocity [m s${}^{-1}$] and $T_{\infty }$ is the free-stream temperature [K] [10,11,12,58,59]. These groups discriminate between two flow regimes:

1. *Subsonic flow* (locally $\hat{M}<1$ or summarily $M_{\infty }≲0.8$), subject to the influence of the downstream pressure, of lower u and often of higher *p*, ρ, *T* and *s*; and
2. *Supersonic flow* (locally $\hat{M}>1$ or summarily $M_{\infty }≳1.2$), which cannot be influenced by the downstream pressure, of higher u and often of lower *p*, ρ, *T* and *s*.

Locally $\hat{M}=1$ is termed *sonic flow*, while summarily $0.8≲M_{\infty }≲1.2$ indicates *transonic flow* and $M_{\infty }≳5$ *hypersonic flow* [59]. Commonly, the Mach number (21) is interpreted by dynamic similarity as the square root of the ratio of inertial to elastic forces [10,12]. Instead of Equation (21), some authors use the Cauchy number $Ca_{\infty }=M_{\infty }^{2}=\rho U_{\infty }^{2}/K$.

Generally, acoustic waves are considered to have a single sonic velocity *a* under given thermodynamic conditions. However, some acoustic waves–such as in bounded systems [3,61,62]–exhibit *wave dispersion*, in which the angular frequency ω [s${}^{-1}$] is a function of the angular wavenumber *k* [m${}^{-1}$], such that the sonic velocity (the *phase celerity*) a=ω/k is a function of frequency [63,64]. If this occurs, interference between different waves will produce wave groups (beats), with the *group celerity* [63,64] and corresponding local group Mach number:

(22) $$\begin{array}{cc} a^{\mathrm{group}} & =\frac{d\omega }{dk}=\frac{d(ak)}{dk}=a+k\frac{da}{dk},\;{\hat{\Pi }}_{a}^{\mathrm{group}}={\hat{M}}^{\mathrm{group}}=\frac{\left||u|\right|}{a^{\mathrm{group}}}, \end{array}$$

For normal wave dispersion, $a^{\mathrm{group}}<a$, da/dk<0 and ${\hat{M}}^{\mathrm{group}}>\hat{M}$. This implies the existence of three distinct information-theoretic flow regimes:

1. *Subsonic flow* (locally $\hat{M}<{\hat{M}}^{\mathrm{group}}<1$), subject to the influence of acoustic waves and wave groups;
2. *Normal mesosonic flow* (locally $\hat{M}<1<{\hat{M}}^{\mathrm{group}}$), influenced by individual acoustic waves but not wave groups; and
3. *Supersonic flow* (locally $1<\hat{M}<{\hat{M}}^{\mathrm{group}}$), which cannot be influenced by acoustic waves or wave groups.

We here use the Greek prefix *meso*- for the “middle” regime. For the transitions, we retain the term *sonic flow* for $\hat{M}=1$, and describe ${\hat{M}}^{\mathrm{group}}=1$ as *group sonic flow*. For the opposite case of anomalous wave dispersion, $a^{\mathrm{group}}>a$, da/dk>0 and ${\hat{M}}^{\mathrm{group}}<\hat{M}$, suggesting the existence of an *anomalous mesosonic flow* regime (locally ${\hat{M}}^{\mathrm{group}}<1<\hat{M}$), influenced by wave groups but not individual waves.

At present, the physical manifestations—if any—of the postulated normal and anomalous mesosonic flow regimes for media with acoustic dispersion are not known. Such effects may be masked by the common use of summary rather than local Mach numbers (21), giving a lumped “transonic” flow with complicated properties. Similarly, the effects of these flow regimes on gradual or sharp flow transitions (such as shock waves) and frictional flow properties are not understood. These phenomena warrant more detailed experimental and theoretical investigation.

#### 4.1.2. Shock Waves

In compressible flows with non-dispersive acoustic waves, it is possible to effect a smooth, isentropic transition between subsonic and supersonic flow (or vice versa) using a nozzle or diffuser, described as a *choke* [10,11,12]. However, the transition from supersonic to subsonic flow is often manifested as a *normal shock wave*, a sharp boundary normal to the flow with discontinuities in u, *p*, ρ, *T* and *s* [59]. From the local entropy production [20] at steady state:

(23) $$\begin{array}{c} {\dot{\sigma }}_{\mathrm{steady}}=\underset{CS}{∯}\left(j_{S}+\rho su\right)\cdot ndA\geq 0 \end{array}$$

where dA is an infinitesimal area element on the control surface CS. Adopting a control volume for a normal shock wave of narrow thickness, with inflow 1, outflow 2 and no non-fluid entropy fluxes ($j_{S}=0$), Equation (23) gives the entropy production per unit area across the shock [J K${}^{-1}$ m${}^{-2}$ s${}^{-1}$] (c.f., [65]):

(24) $$\begin{array}{c} {\overset{˘}{\dot{\sigma }}}_{\mathrm{shock}}=\Delta \left(\rho su\right)\cdot n=\rho_{2}s_{2}u_{2}-\rho_{1}s_{1}u_{1}\geq 0 \end{array}$$

Using relations for *u*, *p*, ρ and *T* derived from the conservation of fluid mass, momentum and energy for inviscid adiabatic steady-state flow across the shock [10,11,17,58,59,66,67,68,69,70,71,72,73], Equation (24) can be rescaled by the internal entropy flux to give the entropic dimensionless group (see Appendix A):

(25) $$\begin{array}{c} \begin{array}{cc} {\overset{˘}{\Pi }}_{\mathrm{shock}}\; & =\frac{{\overset{˘}{\dot{\sigma }}}_{\mathrm{shock}}}{\rho_{1}c_{p}u_{1}}=\frac{s_{2}-s_{1}}{c_{p}}=\ln\left(\frac{\left(2+\left(\gamma -1\right){\hat{M}}_{1}^{2}\right)}{\left(\gamma +1\right){\hat{M}}_{1}^{2}}\right)+\frac{1}{\gamma }\ln\left(\frac{2\gamma {\hat{M}}_{1}^{2}-\gamma +1}{\gamma +1}\right)\\ \mathrm{with}\;\;{\hat{M}}_{2} & =\sqrt{\frac{2+\left(\gamma -1\right){\hat{M}}_{1}^{2}}{2\gamma {\hat{M}}_{1}^{2}-\gamma +1}} \end{array} \end{array}$$

where $c_{p}$ is the specific heat capacity at constant pressure [J K${}^{-1}$ kg${}^{-1}$]. From Equation (25), ${\overset{˘}{\Pi }}_{\mathrm{shock}}>0$ and ${\overset{˘}{\dot{\sigma }}}_{\mathrm{shock}}>0$ for ${\hat{M}}_{1}>1$ and ${\hat{M}}_{2}<1$, so the formation of an entropy-producing normal shock in the transition from supersonic to subsonic flow is permitted by the second law. However, ${\overset{˘}{\Pi }}_{\mathrm{shock}}<0$ and ${\overset{˘}{\dot{\sigma }}}_{\mathrm{shock}}<0$ for ${\hat{M}}_{1}<1$ and ${\hat{M}}_{2}>1$, so the formation of a normal shock in the transition from subsonic to supersonic flow (a rarefaction shock $p_{2}<p_{1}$) is prohibited by the second law (3) (see Appendix A) [10,11,58,59,70,74].

By inwards deflection of supersonic flow (a concave corner), it is also possible to form an *oblique shock wave*, a sharp transition to a different supersonic or subsonic flow with increasing *p*, ρ and *T* [17,59]. This satisfies the same relations for the entropy production in Equations (24) and (25) as a normal shock, but written in terms of velocity components $u_{1}$ and $u_{2}$ normal to the shock, thus with normal Mach components ${\hat{M}}_{n1}={\hat{M}}_{1}\sin\beta$ and ${\hat{M}}_{n2}={\hat{M}}_{2}\sin\left(\beta -\theta \right)$, where β is the shock wave angle and θ is the deflection angle. From the second law ${\overset{˘}{\Pi }}_{\mathrm{shock}}>0$, an oblique shock wave is permissible for ${\hat{M}}_{1}>{\hat{M}}_{2}$, in general with a supersonic transition Mach number, and either two or no β solutions depending on θ [59]. In contrast, outwards deflection of supersonic flow (a convex corner) creates an *expansion fan*, a continuous isentropic transition ${\hat{M}}_{2}>{\hat{M}}_{1}$ with decreasing *p*, ρ and *T* [11,17,59].

The above analysis highlights the confusion in the aerodynamics literature between the specific entropy and the local entropy production. For a non-equilibrium flow system, the second law is defined exclusively by $\dot{\sigma }\geq 0$ in Equation (3) [20], reducing for a sudden transition to ${\overset{˘}{\dot{\sigma }}}_{\mathrm{shock}}\geq 0$ in Equation (24), while the change in specific entropy can (in principle) take any sign $\Delta s=s_{2}-s_{1}⪋0$. From the above analysis, Δs≥0 implies ${\overset{˘}{\dot{\sigma }}}_{\mathrm{shock}}\geq 0$ only for flow transitions satisfying Equation (24) and local continuity $\rho_{1}u_{1}=\rho_{2}u_{2}$; these include normal and oblique shocks. For transitions involving a change in fluid mass flux, or if there are non-fluid entropy fluxes in Equation (24), Δs and ${\overset{˘}{\dot{\sigma }}}_{\mathrm{shock}}$ can have different signs. Similarly, an isentropic process Δs=0 need not indicate zero entropy production ${\overset{˘}{\dot{\sigma }}}_{\mathrm{shock}}=0$. The above analyses are also complicated by fluid turbulence, which generates additional Reynolds entropy flux terms in the local entropy production equation (see analyses in [20,65,75]).

#### 4.1.3. Frictional Compressible Flow

For frictional internal compressible flow with non-dispersive acoustic waves at steady state, the local entropy production (4) reduces to $\hat{\dot{\sigma }}=\nabla \cdot \left(j_{S}+\rho su\right)$ [20]. Assuming one-dimensional adiabatic flow of an ideal gas without chemical or charge diffusion in a conduit of constant cross sections, by the conservation of fluid mass, momentum and energy with friction [11,17,69] and entropic scaling gives the local entropic group (see Appendix B):

(26) $$\begin{array}{c} \begin{array}{cc} {\hat{\Pi }}_{\mathrm{compr}}\left(x\right) & =\frac{\hat{\dot{\sigma }}\left(x\right)\;d_{H}}{\rho_{a}c_{p}a}=\frac{d_{H}}{\rho_{a}c_{p}a}\frac{d\left(\rho (x)s(x)u(x)\right)}{dx}=\frac{d_{H}}{c_{p}}\frac{ds(x)}{dx}\\ & =\frac{2d_{H}\;\left(\gamma -1\right)\left(1-\hat{M}{\left(x\right)}^{2}\right)}{\gamma \left(2+\left(\gamma -1\right)\hat{M}{\left(x\right)}^{2}\right)\;\hat{M}\left(x\right)}\frac{d\hat{M}\left(x\right)}{dx}=d_{H}\;\Theta \left(x\right)\;\frac{d\hat{M}\left(x\right)}{dx}=\frac{1}{2}\left(\gamma -1\right)f\hat{M}{\left(x\right)}^{2} \end{array} \end{array}$$

where *x* is the flow coordinate [m], $d_{H}$ is the pipe hydraulic diameter [m], *f* is the Darcy friction factor [-] and subscript *a* denotes a fluid property at the sonic point. Note the different roles of the specific entropy and the local entropy production. Analysis of the group Θ(x) defined in Equation (26) for γ>1 reveals the following effects of friction:

1. The last term in Equation (26) is positive for all $\hat{M}>0$, hence ${\hat{\Pi }}_{\mathrm{compr}}>0$ and $\hat{\dot{\sigma }}>0$, i.e., the entropy production cannot be zero for finite flow.
2. For subsonic flow $\hat{M}<1$ and Θ>0; hence, the second law ${\hat{\Pi }}_{\mathrm{compr}}>0$ or $\hat{\dot{\sigma }}>0$ implies $d\hat{M}/dx>0$, and so $\hat{M}$ will increase with *x* towards $\hat{M}=1$;
3. For supersonic flow $\hat{M}>1$ and Θ<0; hence, the second law ${\hat{\Pi }}_{\mathrm{compr}}>0$ or $\hat{\dot{\sigma }}>0$ implies $d\hat{M}/dx<0$, and so $\hat{M}$ will decrease with *x* towards $\hat{M}=1$;
4. In both cases, the second law ${\hat{\Pi }}_{\mathrm{compr}}>0$ or $\hat{\dot{\sigma }}>0$ implies ds/dx>0, so the specific entropy *s* will increase with *x* towards $\hat{M}=1$. Integrating Equation (26), this terminates at the maximum specific entropy $s_{a}$;
5. In the sonic limit $\hat{M}\to 1^{\mp }$, Θ→0 and $d\hat{M}/dx\to \pm \infty$, but these limits combine to give $\lim_{\hat{M}\to 1}{\hat{\Pi }}_{\mathrm{compr}}=\frac{1}{2}\left(\gamma -1\right)f>0$ from either direction.

These statements are supported by the plots of $d\hat{M}/dx$, Θ, fluid properties and ${\hat{\Pi }}_{\mathrm{compr}}$ as functions of $\hat{M}$ for the flow of dry air, presented in Appendix B.

The sonic point $x=L^{*}$ and M(x) can then be calculated numerically from the integrated friction equation [10,11,17,69,73]:

(27) $$\begin{array}{c} \begin{array}{c} \frac{f(L^{*}-x)}{d_{H}}=\frac{\gamma +1}{2\gamma }\ln\frac{\left(\gamma +1\right)\hat{M}{\left(x\right)}^{2}}{2+\left(\gamma -1\right)\hat{M}{\left(x\right)}^{2}}+\frac{1-\hat{M}{\left(x\right)}^{2}}{\gamma \hat{M}{\left(x\right)}^{2}} \end{array} \end{array}$$

Flows in conduits longer than $L^{*}$ undergo *frictional choking*, producing a lower subsonic entry Mach number or supersonic flow with a normal shock, so that the flow exits at $\hat{M}=1$ [11,69]. Clearly, such flows are controlled by their entropy production: since they are adiabatic, they cannot export heat, so each fluid element can only achieve a positive local entropy production $\hat{\dot{\sigma }}>0$ by increasing its specific entropy *s* in Equation (26), via permissible changes in *p* and *T*. When *s* reaches its maximum, no solution to Equation (26) with ds/dx>0 is physically realizable, to enable a positive entropy production. This triggers unsteady flow to create the choke. For isothermal flows, flows with heat fluxes, other non-fluid entropy fluxes or chemical reactions, extensions of Equations (26) and (27) are required [10,11,69].

For frictional external compressible flow, the entropy production due to inertial drag and lift can be written in the vector form [20]:

(28) $$\begin{array}{c} {\dot{\sigma }}_{\mathrm{ext},I}^{\mathrm{compr}}=\frac{F_{D}\cdot U}{T_{\infty }}=\frac{\frac{1}{2}\rho_{\infty }A_{s}\;C_{D}\cdot {U\;||U||}^{2}}{T_{\infty }} \end{array}$$

where $F_{D}$ is the drag force [N], U is a representative velocity of the fluid relative to the solid [m s${}^{-1}$], $\rho_{\infty }$ is the free-stream density [kg m${}^{-3}$], $A_{s}$ is the cross-sectional area of the solid [m${}^{2}$] and $C_{D}$ is a vector drag-lift coefficient [-]. Equation (28) can be scaled by sonic conditions to give the entropic group:

(29) $$\begin{array}{c} \Pi_{\mathrm{ext},I}^{\mathrm{compr}}=\frac{{\dot{\sigma }}_{\mathrm{ext},I}^{\mathrm{compr}}}{{\dot{\sigma }}_{\mathrm{ext},I}^{\mathrm{sonic}}}=\frac{\rho_{\infty }\;C_{D}\cdot {M\;||M||}^{2}/T_{\infty }}{\rho_{a}\;C_{Da}/T_{a}}∼C_{D}\cdot {M\;||M||}^{2} \end{array}$$

where ${\dot{\sigma }}_{\mathrm{ext},I}^{\mathrm{sonic}}$ is the sonic inertial entropy production and M is a summary vector Mach number. Generally, the drag coefficient increases significantly beyond a critical Mach number $\left|\right|M_{c}\left|\right|≲1$, due to the local onset of supersonic flow and the formation of shock waves, and then falls to an asymptotic value with increasing ||M||>1 [10,17,59]. In contrast, the lift coefficient of an airfoil exhibits a gradual rise and sudden fall over $\left||M||<|\right|M_{c}\left|\right|<1$, also increasing with the angle of attack [69].

### 4.2. Blast Waves

For chemical combustion in a fluid or solid, the reaction is driven by a *combustion wave* or *blast wave* that moves relative to the reactants at the explosive or detonation velocity $U_{\mathrm{explos}}$ [m s${}^{-1}$]. From Equation (19), this can be scaled by the acoustic velocity measured in the reactants $a_{R}$, giving the information-theoretic group [69]:

(30) $$\begin{array}{c} \Pi_{a_{R}}=M_{\mathrm{explos}}=\frac{U_{\mathrm{explos}}}{a_{R}} \end{array}$$

This defines an explosive Mach number, which discriminates between *detonation* of a high explosive for $M_{\mathrm{explos}}>1$ (typically $M_{\mathrm{explos}}\gg 1)$ in a (compressive) supersonic shock front, or *deflagration* of a low explosive for $M_{\mathrm{explos}}<1$ in a (rarefaction) subsonic flame front [52,69,72].

Explosions in a compressible fluid can be modeled by the one-dimensional conservation equations used for a normal shock in Equation (25), adding the reaction enthalpy and a minimum entropy assumption [69,72,76]. This predicts alternative incoming velocities corresponding to detonation or deflagration; for the former, the outgoing combustion products are expelled at the acoustic velocity relative to the shock front. A kinetic model of detonation (ZND theory) extends this finding, with compression of the reactants at the shock front, causing ignition, heat release and acceleration of the combustion products to the choke point [77,78,79,80].

A large chemical, gas or nuclear explosion in the atmosphere will generate a *spherical shock wave* expanding radially from the source. This provides a famous example of the use of dimensional scaling. Consider a point explosion with shock wave radius *R* [m] governed only by the energy *E* [J], initial density $\rho_{0}$ [kg m${}^{-3}$] and time *t* [s]. Dimensional reasoning gives the self-similar solution $R\propto {\left(Et^{2}/\rho_{0}\right)}^{1/5}$, which with the conservation of mass, momentum and energy for inviscid flow yields power-law relations for *u*, *p*, ρ and *T* with time *t* and radius *r* [52,55,57,70,78,81,82,83]. For short times, these reveal strong heating and near-evacuation of air from the epicenter, and its accumulation behind the shock front.

Surprisingly few authors have examined explosions from an entropic perspective [84,85,86,87,88,89,90], despite its role as their driving force, and the use of minimum [91,92] or maximum [93] entropy closures in some analyses. From Section 4.1, we suggest the use of the local entropic group ${\hat{\Pi }}_{\mathrm{explos}}\left(x\right)$ or ${\hat{\Pi }}_{\mathrm{explos}}\left(r\right)$, extending Equations (26) and (27) to include shock wave, heating and chemical reaction processes.

### 4.3. Pressure Waves

Also related to acoustic waves is the phenomenon of *water hammer*, an overpressure (underpressure) wave in an internal flow of a liquid or gas, caused by rapid closure of a downstream (upstream) valve or pump [12,17]. By reflection at the pipe ends, this causes the cyclic propagation of overpressure and underpressure waves along the pipe, commonly analyzed by the method of characteristics. For flow of an elastic liquid in a thin-walled elastic pipe, the acoustic velocity and the magnitude of the change in pressure are, respectively [12,17,94]:

(31) $$\begin{array}{c} a_{H}=\sqrt{\frac{K}{\rho }{\left(1+\frac{Kd}{E\theta }\left(1-\nu_{p}^{2}\right)\right)}^{-1}},\;|\Delta p|=\rho a_{H}\left|\Delta U\right|, \end{array}$$

where |ΔU| is the magnitude of the change in mean velocity [m s${}^{-1}$], *K* is the bulk elastic modulus of the fluid [Pa], *E* is the elastic modulus of the pipe [Pa], *d* is the pipe diameter [m], θ is the pipe wall thickness [m] and $\nu_{p}$ is Poisson’s ratio for the pipe material [-]. An extended relation is available for gas flows [17]. In liquids, the underpressure wave can cause cavitation (the formation of vapor bubbles), leading to additional shock waves when these collapse at higher pressures [17].

Equations (19) and (31) give the information-theoretic dimensionless group:

(32) $$\begin{array}{c} \Pi_{a_{H}}=Eu_{H}=\frac{\left|\Delta U\right|}{a_{H}}=\frac{\left|\Delta p\right|}{\rho a_{H}^{2}} \end{array}$$

which can be recognized as an Euler number defined for water hammer. By frictional damping in accordance with the Darcy–Weisbach equation [20], the pressure pulse Δp—hence, the wave speed ΔU and the group $\Pi_{a_{H}}$—will also diminish with time.

### 4.4. Stress Waves

Related to acoustic and pressure waves, a variety of waves can occur in solids, liquids and/or along phase boundaries due to the transport of compressive, shear or torsional stresses generated by a sudden failure, expansion or impact. These can be divided into *elastic* or *inelastic waves*, involving reversible or irreversible solid deformation [95,96,97], and also classified into various types of *seismic* or *earthquake waves*. Stress waves can be analyzed by information-theoretic constructs such as Equation (19) to identify the flow regime, but generally are not associated with the mean motion of the medium, so are not examined further here. Blast waves can also be generated in a solid by an explosion or impact, as discussed in Section 4.2.

### 4.5. Surface Gravity Waves

#### 4.5.1. Froude Numbers, Wave Types and Liquid Body Flow Regimes

On the surface of a liquid, energy can be carried by *gravity waves*, involving circular or elliptical rotational oscillations of the fluid in the plane normal to the surface, reducing in scale with depth. These can be classified as *standing waves*, which remain in place, or *progressive waves*, which move across the surface. Using Airy (linear) wave theory, the angular frequency and individual wave (phase) celerity of a two-dimensional progressive surface gravity wave are given by [19,60,98,99,100,101]:

(33) $$\begin{array}{c} \omega^{2}=gk\tanh(ky),\;c_{\mathrm{surf}}=\frac{\omega }{k}=\sqrt{\frac{g}{k}\tanh(ky)}=\sqrt{\frac{\lambda g}{2\pi }\tanh\frac{2\pi y}{\lambda }} \end{array}$$

where λ is the wavelength [m] and *y* is the liquid depth [m]. For an ambient flow with the summary horizontal velocity *U* [m s${}^{-1}$], applying information-theoretic similarity (19) gives the generalized summary Froude number:

(34) $$\begin{array}{c} \Pi_{c_{\mathrm{surf}}}=Fr_{\mathrm{surf}}=\frac{U}{c_{\mathrm{surf}}}=\frac{U}{\sqrt{\frac{g}{k}\tanh(ky)}}=\frac{U}{\sqrt{\frac{\lambda g}{2\pi }\tanh\frac{2\pi y}{\lambda }}} \end{array}$$

A local vector Froude number ${\hat{\mathrm{Fr}}}_{\mathrm{surf}}$ can also be defined based on the local mean velocity $\bar{u}$. However, due to wave dispersion, surface gravity waves generally travel in wave groups. The group celerity–equivalent to the speed of energy transmission [60,99,101]–and the corresponding summary group Froude number are:

(35) $$\begin{array}{c} \begin{array}{cc} c_{\mathrm{surf}}^{\mathrm{group}} & =\frac{d\omega }{dk}=\frac{d(c_{\mathrm{surf}}k)}{dk}=c_{\mathrm{surf}}+k\frac{dc_{\mathrm{surf}}}{dk}=\frac{c_{\mathrm{surf}}}{2}\left(1+\frac{2ky}{\sinh(2ky)}\right),\\ \Pi_{c_{\mathrm{surf}}}^{\mathrm{group}} & =Fr_{\mathrm{surf}}^{\mathrm{group}}=\frac{U}{c_{\mathrm{surf}}^{\mathrm{group}}}=2Fr_{\mathrm{surf}}{\left(1+\frac{2ky}{\sinh(2ky)}\right)}^{-1} \end{array} \end{array}$$

Curiously, the two Froude numbers in Equations (34) and (35) are not in common use. Variants of the celerities in Equations (33)–(35) are available for gravity waves on the interface between two liquids [100,102]. Since 0<x/sinh(x)≤1 for x=2ky>0, surface gravity waves usually exhibit *normal dispersion* $c_{\mathrm{surf}}^{\mathrm{group}}<c_{\mathrm{surf}}$ and $dc_{\mathrm{surf}}/dk<0$, with individual waves advancing faster than the group. The exception in the limit ky→0 is examined below.

Usually, three cases of surface gravity waves are distinguished:

1. For *deepwater (deep liquid)* or *short waves*: ky≳π or λ/y≲2; thus, tanh(ky)→1 in Equation (33), hence [19,60,83,98,99,100,103]:
  (36) $$\begin{array}{c} c_{\lambda }=\sqrt{\frac{g}{k}}=\sqrt{\frac{\lambda g}{2\pi }},\;\Pi_{c_{\mathrm{surf}}}\to Fr_{\lambda }=\frac{U}{c_{\lambda }}=U\sqrt{\frac{k}{g}}=U\sqrt{\frac{2\pi }{\lambda g}} \end{array}$$
  Such waves move freely by circular motions of the fluid, with little net horizontal transport. Deep waves travel in wave groups: in the deepwater limit ky→∞, 2ky/sinh(2ky)→0 in Equation (35), giving the group celerity $c_{\lambda }^{\mathrm{group}}=\frac{1}{2}c_{\lambda }$ and group Froude number $Fr_{\lambda }^{\mathrm{group}}=2Fr_{\lambda }$. Despite their simplicity, neither $Fr_{\lambda }^{\mathrm{group}}$ nor $Fr_{\lambda }$ are in common use. For wave drag on a ship, the Froude number $Fr_{\mathrm{ship}}=U/\sqrt{gL}$ is used, where *U* is the ship velocity [m s${}^{-1}$] and *L* is the ship length [m] [10,18].
2. For *transitional waves*: π/10≲ky≲π or 2≲λ/y≲20, the wave motion is impeded by contact with the bottom, producing elliptical motions of the fluid. Such waves form in natural water bodies by the shoaling of deepwater waves as they approach the shoreline. The generalized phase celerity and Froude number (33) and (34), and the generalized group celerity and Froude number (35), apply. More complicated (nonlinear) wave descriptions can also be used, including *Stokesian waves* for λ/y≲10, a superposition of cosine wave forms, and *cnoidal waves* for λ/y≳10, comprising horizontally asymmetric waveforms with pointed crests [98].
3. For *shallow* or *long waves*: ky≲π/10 or λ/y≳20; thus, tanh(ky)→ky in Equation (33), giving [10,12,19,60,83,100,101,103,104]:
  (37) $$\begin{array}{c} c_{y}=\sqrt{gy},\;\Pi_{c_{\mathrm{surf}}}\to Fr_{y}=\frac{U}{\sqrt{gy}} \end{array}$$
  In the shallow limit, ky→0, sinh(2ky)→2ky and $Fr_{y}^{\mathrm{group}}\to Fr_{y}$ in Equation (35), so there is no separate group celerity (producing *non-dispersive waves*). Equation (37) is applied to open channel flows with rectangular cross sections. For channels of low slope and arbitrary cross sections (of low aspect ratio), Equation (37) is commonly generalized as [10,19,104,105]:
  (38) $$\begin{array}{c} c_{y_{h}}=\sqrt{gy_{h}},\;\Pi_{c_{\mathrm{surf}}}\to Fr_{y_{h}}=\frac{U}{\sqrt{gy_{h}}} \end{array}$$
  where $y_{h}=A/B$ is the hydraulic mean depth [m], *A* is the channel cross-sectional area [m${}^{2}$] and *B* is the channel top width [m]. For a rectangular channel, $y_{h}=y$.

Steady incompressible open channel flows generally satisfy the conditions for shallow waves, which communicate the occurrence of a downstream influence (such as a sudden obstruction). The Froude number in Equation (37) or (38) then discriminates between two flow regimes [10,12,104]:

1. *Subcritical flow* ($Fr_{y}$ or $Fr_{y_{h}}<1$), subject to the influence of downstream obstructions, of lower velocity *U* and higher water height *y*; and
2. *Supercritical flow* ($Fr_{y}$ or $Fr_{y_{h}}>1$), which cannot be influenced by downstream obstructions, of higher velocity *U* and lower water height *y*.

Locally $Fr_{y}$ or $Fr_{y_{h}}=1$ is termed *critical flow*, occurring at the *critical depth* $y_{c}$ [m].

The above flow regimes can be extended to flows with deepwater or transitional waves, but the analysis must take into account the effect of wave dispersion, which produces two different Froude numbers $Fr_{\mathrm{surf}}$ and $Fr_{\mathrm{surf}}^{\mathrm{group}}$ (Equations (34) and (35), respectively), for individual waves and wave groups. By normal wave dispersion, $c_{\mathrm{surf}}^{\mathrm{group}}<c_{\mathrm{surf}}$ and $Fr_{\mathrm{surf}}^{\mathrm{group}}>Fr_{\mathrm{surf}}$. This creates the possibility of three information-theoretic flow regimes:

1. *Subcritical flow* ($Fr_{\mathrm{surf}}<Fr_{\mathrm{surf}}^{\mathrm{group}}<1$), subject to the influence of surface gravity waves and wave groups;
2. *Normal mesocritical flow* ($Fr_{\mathrm{surf}}<1<Fr_{\mathrm{surf}}^{\mathrm{group}}$), influenced by individual surface gravity waves but not wave groups; and
3. *Supercritical flow* ($1<Fr_{\mathrm{surf}}<Fr_{\mathrm{surf}}^{\mathrm{group}}$), which cannot be influenced by surface gravity waves or wave groups.

We retain the term *critical flow* for $Fr_{\mathrm{surf}}=1$, and describe $Fr_{\mathrm{surf}}^{\mathrm{group}}=1$ as *group critical flow*. The physical manifestations of the postulated mesocritical flow regime in systems with deepwater or transitional waves are not known, and may again be masked by the common use of summary (Equation (34)) rather than local Froude numbers. These systems warrant more detailed experimental and theoretical investigation. Deep liquid bodies will also be influenced by internal gravity waves, examined in Section 4.7.

#### 4.5.2. Hydraulic Jumps in Open Channel Flow

In many open channel flows, it is possible to effect a smooth transition between subcritical and supercritical flow (or vice versa) using a pinched channel (Venturi flume) or stepped bed, described as a *choke* [19,98,104]. However, the transition from supercritical to subcritical flow is often manifested as an *hydraulic jump*, with sharp changes in *U* and *y*. For a macroscopic control volume extending across a jump in a rectangular channel, with inflow 1, outflow 2 and no non-fluid entropy fluxes, by the conservation of mass and momentum with energy loss [11,19,98,104,105], the total entropy production is:

(39) $$\begin{array}{c} \begin{array}{cc} {\dot{\sigma }}_{\mathrm{jump}} & =\frac{\rho gQ\Delta E}{T}=\frac{\rho gQ}{T}\frac{{\left(y_{2}-y_{1}\right)}^{3}}{4y_{1}y_{2}}\geq 0\\ \mathrm{with}\;\frac{y_{2}}{y_{1}} & =\frac{1}{2}\left(\sqrt{1+8Fr_{y_{1}}^{2}}-1\right)=2{\left(\sqrt{1+8Fr_{y_{2}}^{2}}-1\right)}^{-1} \end{array} \end{array}$$

where *Q* is the volumetric flow rate [m${}^{3}$ s${}^{-1}$] and ΔE is the loss in energy per unit weight [J N${}^{-1}$ = m]. Scaling by the entropy flow rate gives the entropic group:

(40) $$\begin{array}{c} \Pi_{\mathrm{jump}}=\frac{{\dot{\sigma }}_{\mathrm{jump}}}{\rho c_{p}Q}=\frac{g\Delta E}{c_{p}T}=\frac{g}{c_{p}T}\frac{{\left(y_{2}-y_{1}\right)}^{3}}{4y_{1}y_{2}}\geq 0 \end{array}$$

From Equations (39) and (40), $\Pi_{\mathrm{jump}}>0$ and ${\dot{\sigma }}_{\mathrm{jump}}>0$ for $Fr_{y_{1}}>1$ and $Fr_{y_{2}}<1$, so the formation of an entropy-producing hydraulic jump in the transition from supercritical to subcritical flow is permitted by the second law. However, $\Pi_{\mathrm{jump}}<0$ and ${\dot{\sigma }}_{\mathrm{jump}}<0$ for $Fr_{y_{1}}<1$ and $Fr_{y_{2}}>1$, so the formation of an hydraulic jump in the transition from subcritical to supercritical flow (a reverse jump $y_{2}<y_{1}$) is prohibited by the second law (see Appendix C).

By the inward deflection of supercritical flow or by interaction with wall boundaries, it is also possible to form an *oblique hydraulic jump*, a sharp transition to a different supercritical or subcritical flow at the angle β∈(0,π) to the flow centerline [98]. This satisfies the same relations for the entropy production in Equations (39) and (40) as a normal jump, but written in terms of the velocities $U_{1}$ and $U_{2}$ normal to the jump, thus with normal Froude numbers $Fr_{y,ni}=Fr_{yi}\sin\beta$ for i∈{1,2} [98]. From the second law $\Pi_{\mathrm{jump}}>0$, an oblique jump is permissible for $Fr_{y1}>{\left(\sin\beta \right)}^{-1}>Fr_{y2}$, so in general with the transitional Froude number $Fr_{yc}={\left(\sin\beta \right)}^{-1}\geq 1$.

#### 4.5.3. Frictional Gradually Varied Open Channel Flow

Frictional open channel flows at a steady state can be classified as (i) *uniform flows* of constant water elevation $y=y_{0}$, due to the equilibrium between frictional and gravitational forces in a long channel; (ii) *gradually-varied flows*, with a smooth flow profile y(x), where *x* is the flow direction [m]; or (iii) *rapidly-varied flows*, with a sharp change in y(x) in response to a sudden constriction [98,104,105]. The resistance for arbitrary cross sections is often represented by the Manning equation $U=R_{H}^{2/3}\sqrt{S}/n$, where $R_{H}=A/P_{w}$ is the hydraulic radius [m], *A* is the channel cross-sectional area [m${}^{2}$], $P_{w}$ is the wetted perimeter [m], *n* is Manning’s constant for the channel type [s m${}^{-1/3}$] and $S=dH_{L}/dx$ is the hydraulic slope [-], in which $H_{L}$ is the head loss [m] and *x* is the horizontal coordinate [m] [19,98,104,105]. The entropy production per unit channel length [J K${}^{-1}$ m${}^{-1}$ s${}^{-1}$] by inertial dispersion is [20,50,106,107,108,109]:

(41) $$\begin{array}{c} {\tilde{\dot{\sigma }}}_{\mathrm{open}}\left(x\right)=\frac{\rho gQ}{T}\frac{dH_{L}}{dx}=\frac{\rho gSQ}{T}=\frac{\rho gQ^{3}n^{2}P_{w}^{4/3}}{A^{10/3}T} \end{array}$$

For a rectangular channel of bed slope $S_{0}$ [-], constant width *B* and constant flow rate Q=qB, where q=Uy is the flow rate per unit width [m${}^{2}$ s${}^{-1}$], substituting A=By, $P_{w}=B+2y$, $Fr_{y}=q/\left(y^{3/2}\sqrt{g}\right)$ and the energy equation into Equation (41) and rescaling gives the local entropic group (see Appendix D):

(42) $$\begin{array}{c} \begin{array}{cc} {\tilde{\Pi }}_{\mathrm{open}}\left(x\right)\; & =\frac{{\tilde{\dot{\sigma }}}_{\mathrm{open}}\left(x\right)}{\rho c_{p}q}=\frac{gBS(x)}{c_{p}T}=\frac{gBS_{0}\left(x\right)}{c_{p}T}+\frac{gB(S\left(x\right)-S_{0}\left(x\right))}{c_{p}T}\\ & =\frac{gBS_{0}\left(x\right)}{c_{p}T}+\frac{gB\left(Fr_{y}{\left(x\right)}^{2}-1\right)}{c_{p}T}\frac{dy(x)}{dx}\\ & =\frac{gBS_{0}\left(x\right)}{c_{p}T}-\frac{2B\left(Fr_{y}{\left(x\right)}^{2}-1\right){\left(gq\right)}^{2/3}}{3c_{p}T\;Fr_{y}{\left(x\right)}^{5/3}}\frac{dFr_{y}\left(x\right)}{dx}=\frac{q^{2}g\;n^{2}{\left(B+2y(x)\right)}^{4/3}}{c_{p}T\;y{\left(x\right)}^{10/3}\;B^{1/3}} \end{array} \end{array}$$

Analysis of Equation (42) reveals the following effects of friction:

1. The last term in Equation (42) is positive for all $Fr_{y}>0$ and y>0, hence ${\tilde{\Pi }}_{\mathrm{open}}>0$ and ${\tilde{\dot{\sigma }}}_{\mathrm{open}}>0$, i.e., the entropy production cannot be zero for finite flow.
2. In contrast to frictional compressible flows (Section 4.1.3), frictional open channel flows are subject to a larger set of upstream and downstream boundary conditions. These, in combination with the channel slope, flow rate and flow regime—under the constraint of a positive entropy production—determine the flow profile y(x) that will be realized. Some profiles terminate or start at the critical depth $y=y_{c}$, at which $Fr_{y_{h}}=1$; some at the uniform depth $y=y_{0}$, at which friction and gravity are in equilibrium; some start from a (theoretical) zero depth y=0; and some terminate in a horizontal water surface [19,98,104,105].
3. For subcritical flow $Fr_{y}<1$ and $y>y_{c}$, from the second law ${\tilde{\Pi }}_{\mathrm{open}}>0$ or ${\tilde{\dot{\sigma }}}_{\mathrm{open}}>0$ in Equation (42):
  (a) For uniform flow $S=S_{0}$, Equation (42) implies constant $y=y_{0}$ and $Fr_{y}=Fr_{y0}$;
  (b) For $S_{0}<S$, Equation (42) implies dy/dx<0 and $dFr_{y}/dx>0$, so $Fr_{y}$ will increase with *x*, while y(x) will decrease with *x* (a drawdown curve);
  (c) For $0<S<S_{0}$, Equation (42) implies dy/dx>0 and $dFr_{y}/dx<0$, so $Fr_{y}$ will decrease with *x*, while y(x) will increase with *x* (a backwater curve).
4. For supercritical flow $Fr_{y}>1$ and $y<y_{c}$, from the second law ${\tilde{\Pi }}_{\mathrm{open}}>0$ or ${\tilde{\dot{\sigma }}}_{\mathrm{open}}>0$ in Equation (42):
  (a) For uniform flow $S=S_{0}$, Equation (42) implies constant $y=y_{0}$ and $Fr_{y}=Fr_{y0}$;
  (b) For $S_{0}<S$, Equation (42) implies dy/dx>0 and $dFr_{y}/dx<0$; hence, $Fr_{y}$ will decrease with *x*, while y(x) will increase with *x* (a backwater curve);
  (c) For $0<S<S_{0}$, Equation (42) implies dy/dx<0 and $dFr_{y}/dx>0$; hence, $Fr_{y}$ will increase with *x*, while y(x) will decrease with *x* (a drawdown curve);
5. In the critical limit $Fr_{y}\to 1^{\mp }$ and $y\to y_{c}^{\pm }$, $(Fr_{y}^{2}-1)\to 0$ and dy/dx→∓∞, but these limits combine to give $\lim_{Fr_{y}\to 1}{\tilde{\Pi }}_{\mathrm{open}}=q^{2}g\;n^{2}{\left(B+2y_{c}\right)}^{4/3}/c_{p}T\;y_{c}^{10/3}B^{1/3}>0$ from either direction. A special case of critical uniform flow ($y=y_{0}=y_{c}$ and $Fr_{y}=Fr_{y0}=1$) can form, but otherwise, critical flow will occur as a limiting case at the position $x=x_{c}$.

These statements are supported by the plots of *y* and ${\tilde{\Pi }}_{\mathrm{open}}$ as functions of $Fr_{y}$ for a worked example of open channel flow, presented in Appendix D.

For gradually varied flows, the flow profile y(x) and $Fr_{y}\left(x\right)$ can be calculated by numerical integration of the friction equation in (42) [19,98,104,105]. For profiles terminating at $y_{c}$, if the channel is longer than the critical length $x_{c}$, the flow will undergo a process similar to *frictional choking* (Section 4.1) to enable it to pass through $Fr_{y}=1$. Such flows are controlled by their entropy production: each cross-sectional fluid element can only achieve a positive entropy production ${\tilde{\dot{\sigma }}}_{\mathrm{open}}>0$ by altering its depth in accordance with an individual flow profile, as defined in items (3)–(4) above. When y(x) reaches the critical depth $y_{c}$, at which $\left(Fr_{y}^{2}-1\right)$ changes sign, no solution to Equation (42) along that profile with the same sign of dy/dx is physically realizable, consistent with a positive entropy production. This triggers the choke, manifested as a transition to a different flow profile (commonly, a smooth transition from subcritical to supercritical flow, or an hydraulic jump). For different flow sections, changes in slope, channels of variable width or spatially-varied flow rates, extensions of Equation (42) are required [98,104,105].

### 4.6. Surface Gravity–Capillary Waves

Energy can also be carried by *surface capillary waves* held by surface tension on a gas–liquid surface. The angular frequency, phase celerity and group celerity of mixed transitional surface gravity–capillary waves are [3,98,99,100]:

(43) $$\begin{array}{c} \begin{array}{cc} \omega^{2} & =k\left(g+\frac{\varsigma k^{2}}{\rho }\right)\tanh\left(ky\right),\;c_{\mathrm{surf}}=\frac{\omega }{k}=\sqrt{\left(\frac{g}{k}+\frac{\varsigma k}{\rho }\right)\tanh(ky)},\;\\ c_{\mathrm{surf}}^{\mathrm{group}}\; & =\frac{d\omega }{dk}=\frac{d(c_{\mathrm{surf}}k)}{dk}=c_{\mathrm{surf}}+k\frac{dc_{\mathrm{surf}}}{dk}=\frac{c_{\mathrm{surf}}}{2}\left(\frac{3\varsigma k^{2}+\rho g}{\varsigma k^{2}+\rho g}+\frac{2ky}{\sinh(2ky)}\right) \end{array} \end{array}$$

where ς is the surface or interfacial tension [J m${}^{-2}$]. These can be expressed in terms of a wave Eötvös or Bond number $Bo=\rho g/\varsigma k^{2}=\rho g\lambda^{2}/4\pi \varsigma$ (compare [20]), to give the corresponding Froude numbers (19):

(44) $$\begin{array}{c} \begin{array}{cc} \Pi_{c_{\mathrm{surf}}} & =Fr_{\mathrm{surf}}=\frac{U}{c_{\mathrm{surf}}}=\frac{U}{\sqrt{\frac{\varsigma k}{\rho }\left(Bo+1\right)\tanh\left(ky\right)}}=\frac{U}{\sqrt{\frac{g}{k}\left({Bo}^{-1}+1\right)\tanh\left(ky\right)}}\\ \Pi_{c_{\mathrm{surf}}}^{\mathrm{group}} & =Fr_{\mathrm{surf}}^{\mathrm{group}}=\frac{U}{c_{\mathrm{surf}}^{\mathrm{group}}}=\frac{U}{\frac{c_{\mathrm{surf}}}{2}\left(\frac{Bo+3}{Bo+1}+\frac{2ky}{\sinh(2ky)}\right)} \end{array} \end{array}$$

These have two sets of limits:

1. *Deepwater waves* for ky→∞ hence tanh(ky)→1 and 2ky/sinh(2ky)→0, or *shallow waves* for ky→0 hence tanh(ky)→ky and sinh(2ky)→2ky (see Section 4.5); and
2. *Pure surface gravity waves* (Equations (33)–(35)) for Bo→∞, or *pure capillary waves* for Bo→0.

For deepwater pure capillary waves, the phase celerity [83,100,103] and Froude number are obtained as:

(45) $$\begin{array}{c} c_{\lambda (\varsigma )}=\sqrt{\frac{\varsigma k}{\rho }}=\sqrt{\frac{2\pi \varsigma }{\rho \lambda }},\;\Pi_{c_{\lambda (\varsigma )}}=Fr_{\lambda (\varsigma )}=\frac{U}{c_{\lambda (\varsigma )}}=U\sqrt{\frac{\rho }{\varsigma k}}=U\sqrt{\frac{\rho \lambda }{2\pi \varsigma }} \end{array}$$

The group celerity and Froude number are $c_{\lambda (\varsigma )}^{\mathrm{group}}=\frac{3}{2}c_{\lambda (\varsigma )}$ [83] and $Fr_{\lambda (\varsigma )}^{\mathrm{group}}=\frac{2}{3}Fr_{\lambda (\varsigma )}$. In contrast, for shallow pure capillary waves:

(46) $$\begin{array}{c} c_{y(\varsigma )}=\sqrt{\frac{y\varsigma k^{2}}{\rho }}=2\pi \sqrt{\frac{y\varsigma }{\rho \;\lambda^{2}}},\;\Pi_{c_{y(\varsigma )}}=Fr_{y(\varsigma )}=\frac{U}{c_{y(\varsigma )}}=U\sqrt{\frac{\rho }{y\varsigma k^{2}}}=\frac{U}{2\pi }\sqrt{\frac{\rho \lambda^{2}}{y\varsigma }} \end{array}$$

with the group celerity $c_{y(\varsigma )}^{\mathrm{group}}=2c_{y(\varsigma )}$ and group Froude number $Fr_{y(\varsigma )}^{\mathrm{group}}=\frac{1}{2}Fr_{y(\varsigma )}$. As evident, flows with pure capillary waves exhibit *anomalous dispersion*, with individual waves advancing more slowly than the group.

Due to the occurrence of two Froude numbers with normal or anomalous dispersion, it is possible for flows with surface gravity–capillary waves to exhibit four different information-theoretic flow regimes (compare Section 4.5.1):

1. *Subcritical flow* ($\{Fr_{\mathrm{surf}},Fr_{\mathrm{surf}}^{\mathrm{group}}\}<1$), subject to the influence of waves and wave groups;
2. *Normal mesocritical flow* ($Fr_{\mathrm{surf}}<1<Fr_{\mathrm{surf}}^{\mathrm{group}}$) for systems with normal dispersion, influenced by individual waves but not wave groups;
3. *Anomalous mesocritical flow* ($Fr_{\mathrm{surf}}^{\mathrm{group}}<1<Fr_{\mathrm{surf}}$) for systems with anomalous dispersion, influenced by wave groups but not individual waves; and
4. *Supercritical flow* ($1<\{Fr_{\mathrm{surf}},Fr_{\mathrm{surf}}^{\mathrm{group}}\}$), which cannot be influenced by waves or wave groups.

The existence of the mesocritical or anomalous mesocritical flow regime will depend on the dominance of gravity or capillary waves and the liquid depth. The crossover point, at which both mesocritical regimes vanish, is defined by $c_{\mathrm{surf}}=c_{\mathrm{surf}}^{\mathrm{group}}$ in Equation (43) or $Fr_{\mathrm{surf}}=Fr_{\mathrm{surf}}^{\mathrm{group}}$ in Equation (44), thus along the curve described by the wave Bond number:

(47) $$\begin{array}{c} Bo=\frac{\sinh(2ky)+2ky}{\sinh(2ky)-2ky} \end{array}$$

The physical manifestations of the postulated normal and anomalous mesocritical flow regimes in systems with gravity–capillary waves are not known, and warrant more detailed experimental investigation.

### 4.7. Internal Gravity Waves

Related to surface waves are *internal (gravity) waves*, transverse waves within a density-stratified fluid, including within the oceans due to temperature and/or salinity variations, or in the atmosphere due to pressure and temperature gradients [101,110]. In the atmosphere, internal waves are often revealed by stationary (lenticular) clouds or repeating cloud patterns (herringbone or mackerel sky) [111].

For a vertically stratified fluid with horizontal and vertical coordinates $x={\left[x,z\right]}^{⊤}$ in the plane of internal wave motion, under the Boussinesq approximation of small changes in density (which excludes sound waves), the frequency and celerities of individual waves and wave groups are [60,100,101,102,110,112,113,114,115,116,117,118,119]:

(48) $$\begin{array}{c} \begin{array}{cccc} \omega^{2} & =\frac{N_{0}^{2}k_{x}^{2}}{{\left||k|\right|}^{2}},\; & \mathrm{with}\;N_{0}^{2} & ≃-\frac{g}{\rho_{0}}\frac{d\rho }{dz}≃\frac{g}{T_{0}}\left(\frac{dT}{dz}+\Gamma \right)\\ c_{\mathrm{int}}\; & =\frac{\omega k}{{\left||k|\right|}^{2}}=\pm \frac{N_{0}k_{x}k}{{\left||k|\right|}^{3}},\; & c_{\mathrm{int}}^{\mathrm{group}}\; & =\nabla_{k}\omega =\pm \frac{N_{0}k_{z}Rk}{{\left||k|\right|}^{3}}=\pm \frac{N_{0}k_{z}}{{\left||k|\right|}^{3}}\left[\begin{array}{c} k_{z}\\ -k_{x} \end{array}\right] \end{array} \end{array}$$

where ω is the intrinsic angular frequency (moving with the flow) [s${}^{-1}$], $k={\left[k_{x},k_{z}\right]}^{⊤}$ is the vector wavenumber [m${}^{-1}$] in the plane of individual wave migration, $N_{0}$ is the Brunt–Väisälä or buoyancy frequency [s${}^{-1}$], a characteristic frequency of the stratified fluid, $\Gamma =g\left(\gamma -1\right)/R^{*}\gamma$ is the adiabatic lapse rate [K m${}^{-1}$], $R=\left(\begin{array}{cc} 0 & 1\\ -1 & 0 \end{array}\right)$ is a 90-degree clockwise rotation matrix, and subscript 0 indicates a reference value. Equation (48) gives the intrinsic phase and group celerities (expressed relative to the medium) [119], consistent with the treatment of other wave systems (e.g., Section 4.1 or Section 4.5). Some authors (e.g., [113,114,116]) base their formulation on the absolute frequency and absolute celerities (expressed relative to a fixed frame of reference); this is not used here. A number of authors (e.g., [113,114,117]) use directional phase speeds that do not constitute a vector, whereas the phase celerity $c_{\mathrm{int}}$ in Equation (48) is correctly defined as a vector (see Appendix E). The first form of $N_{0}$ in Equation (48) applies to a density-stratified liquid such as a saline ocean, and the second to the atmosphere or a temperature-stratified ocean. For fluids with two gradients, a composite relation for $N_{0}$ may be needed [118]. Other celerity relations can be derived for different assumptions, such as non-uniformly stratified fluids, non-Boussinesq fluids or nonlinear waves [101,117].

Since the celerities in Equation (48) are multidimensional, the wave dispersion is now defined by $c_{\mathrm{int}}^{\mathrm{group}}-c_{\mathrm{int}}$, representing changes in both magnitude and direction. For the change in magnitude, component-wise analysis of the two celerities gives a generic multidimensional dispersion relation (A24) (see Appendix F). From this, Equation (48) gives:

(49) $$\begin{array}{c} c_{\mathrm{int}}^{\mathrm{group}}-c_{\mathrm{int}}=\mp \frac{N_{0}}{{\left||k|\right|}^{3}}\left[\begin{array}{c} k_{x}^{2}-k_{z}^{2}\\ 2k_{x}k_{z} \end{array}\right] \end{array}$$

A curious feature of Equation (48) is that the two celerities are orthogonal, $c_{\mathrm{int}}\cdot c_{\mathrm{int}}^{\mathrm{group}}\propto k^{⊤}Rk=0$. In consequence, internal gravity wave groups (and the wave energy) propagate normal to the individual waves, in alignment with the wave crests and troughs [100,101,102,114,115,116,117,119].

For flow with the mean local velocity $\bar{u}={\left[\bar{u},\bar{w}\right]}^{⊤}$, several definitions of information-theoretic similarity are available in Equations (19) and (20). However, the directional form ${\hat{\Pi }}_{\mathrm{info}}=u\cdot n/c\cdot n$ is affected by singularities at c·n=0. For flows with internal waves of fixed wavenumber and direction k, the component-wise formulation in Equation (20) is instructive, giving the local vector phase and group Froude numbers:

(50) $$\begin{array}{c} \begin{array}{cc} {\tilde{\Pi }}_{c_{\mathrm{int}}}\; & ={\tilde{\mathrm{Fr}}}_{c_{\mathrm{int}}}=\bar{u}⊘c_{\mathrm{int}}=\frac{{\left||k|\right|}^{2}}{\omega }\;\bar{u}⊘k=\pm \frac{{\left||k|\right|}^{3}}{N_{0}k_{x}}\;\bar{u}⊘k=\pm \frac{{\left||k|\right|}^{3}}{N_{0}k_{x}}\;\left[\begin{array}{c} \bar{u}/k_{x}\\ \bar{w}/k_{z} \end{array}\right]\\ {\tilde{\Pi }}_{c_{\mathrm{int}}}^{\mathrm{group}}\; & ={\tilde{\mathrm{Fr}}}_{c_{\mathrm{int}}}^{\mathrm{group}}=\bar{u}⊘c_{\mathrm{int}}^{\mathrm{group}}=\bar{u}⊘\nabla_{k}\omega =\pm \frac{{\left||k|\right|}^{3}}{N_{0}k_{z}}\;\bar{u}⊘Rk=\pm \frac{{\left||k|\right|}^{3}}{N_{0}k_{z}}\;\left[\begin{array}{c} \bar{u}/k_{z}\\ -\bar{w}/k_{x} \end{array}\right] \end{array} \end{array}$$

For flows with internal waves of fixed wavenumber and arbitrary direction ||k||, the vector formulation in Equation (19) gives:

(51) $$\begin{array}{c} \begin{array}{cc} {\hat{\Pi }}_{c_{\mathrm{int}}}\; & ={\hat{\mathrm{Fr}}}_{c_{\mathrm{int}}}=\frac{\bar{u}}{\left|\right|c_{\mathrm{int}}\left|\right|}=\frac{{\left||k|\right|}^{2}\;\bar{u}}{N_{0}k_{x}}\\ {\hat{\Pi }}_{c_{\mathrm{int}}}^{\mathrm{group}}\; & ={\hat{\mathrm{Fr}}}_{c_{\mathrm{int}}}^{\mathrm{group}}=\frac{\bar{u}}{\left|\right|c_{\mathrm{int}}^{\mathrm{group}}\left|\right|}=\frac{{\left||k|\right|}^{2}\;\bar{u}}{N_{0}k_{z}} \end{array} \end{array}$$

Equations (50) and (51) do not appear to have been defined previously. As evident, the horizontal components of ${\tilde{\mathrm{Fr}}}_{c_{\mathrm{int}}}$ and ${\tilde{\mathrm{Fr}}}_{c_{\mathrm{int}}}^{\mathrm{group}}$ depend on the relative sizes of $k_{x}$ and $k_{z}$, while the vertical components are of the same magnitude but opposite sign. In contrast, ${\hat{\mathrm{Fr}}}_{c_{\mathrm{int}}}$ and ${\hat{\mathrm{Fr}}}_{c_{\mathrm{int}}}^{\mathrm{group}}$ differ only in the denominator terms $k_{x}$ or $k_{z}$. Both definitions require $k_{x},k_{z}\neq 0$.

The vector forms of Equations (50) and (51) and the occurrence of two Froude numbers in each formulation suggest the existence of four vectorial information-theoretic flow regimes for internal gravity waves, as follows:

1. *Subcritical flow* ($\left|\right|{\tilde{\mathrm{Fr}}}_{c_{\mathrm{int}}}\left||,|\right|{\tilde{\mathrm{Fr}}}_{c_{\mathrm{int}}}^{\mathrm{group}}\left|\right|<1$ or $\left|\right|{\hat{\mathrm{Fr}}}_{c_{\mathrm{int}}}\left||,|\right|{\hat{\mathrm{Fr}}}_{c_{\mathrm{int}}}^{\mathrm{group}}\left|\right|<1$), subject to the influence of waves and wave groups;
2. *Normal mesocritical flow* ($\left|\right|{\tilde{\mathrm{Fr}}}_{c_{\mathrm{int}}}\left||<1<|\right|{\tilde{\mathrm{Fr}}}_{c_{\mathrm{int}}}^{\mathrm{group}}\left|\right|$ or $\left|\right|{\hat{\mathrm{Fr}}}_{c_{\mathrm{int}}}\left||<1<|\right|{\hat{\mathrm{Fr}}}_{c_{\mathrm{int}}}^{\mathrm{group}}\left|\right|$) with normal dispersion, influenced by individual waves but not wave groups;
3. *Anomalous mesocritical flow* ($\left|\right|{\tilde{\mathrm{Fr}}}_{c_{\mathrm{int}}}^{\mathrm{group}}\left||<1<|\right|{\tilde{\mathrm{Fr}}}_{c_{\mathrm{int}}}\left|\right|$ or $\left|\right|{\hat{\mathrm{Fr}}}_{c_{\mathrm{int}}}^{\mathrm{group}}\left||<1<|\right|{\hat{\mathrm{Fr}}}_{c_{\mathrm{int}}}\left|\right|$) with anomalous dispersion, influenced by wave groups but not individual waves; and
4. *Supercritical flow* ($1<||{\tilde{\mathrm{Fr}}}_{c_{\mathrm{int}}}\left||,|\right|{\tilde{\mathrm{Fr}}}_{c_{\mathrm{int}}}^{\mathrm{group}}\left|\right|$ or $1<||{\hat{\mathrm{Fr}}}_{c_{\mathrm{int}}}\left||,|\right|{\hat{\mathrm{Fr}}}_{c_{\mathrm{int}}}^{\mathrm{group}}\left|\right|$), which cannot be influenced by waves or wave groups.

These extend the postulated regimes for deepwater and transitional surface gravity waves (Section 4.5.1) and surface gravity–capillary waves (Section 4.6), to account for the vector forms of the velocity and celerity fields. The physical manifestations of the postulated normal and anomalous mesocritical flow regimes—and their effect on flow transitions—are not known, and warrant further experimental investigation.

From Equation (51), ${\hat{\mathrm{Fr}}}_{c_{\mathrm{int}}}$ and ${\hat{\mathrm{Fr}}}_{c_{\mathrm{int}}}^{\mathrm{group}}$ are both aligned with the local mean velocity vector u, in keeping with previous definitions of vector Mach or Froude numbers. In contrast, from Equation (50), ${\tilde{\mathrm{Fr}}}_{c_{\mathrm{int}}}$ and ${\tilde{\mathrm{Fr}}}_{c_{\mathrm{int}}}^{\mathrm{group}}$ are in general not orthogonal nor parallel nor aligned with u. Instead, from the definition of the dot product, they are separated by the angle θ in:

(52) $$\begin{array}{c} \cos\theta =\pm \frac{|k_{x}\left|\;\right|k_{z}|\left({\bar{u}}^{2}-{\bar{w}}^{2}\right)}{\sqrt{\left({\bar{u}}^{2}{k_{x}}^{2}+{\bar{w}}^{2}{k_{z}}^{2}\right)\;\left({\bar{u}}^{2}{k_{z}}^{2}+{\bar{w}}^{2}{k_{x}}^{2}\right)}} \end{array}$$

**Figure 1:**
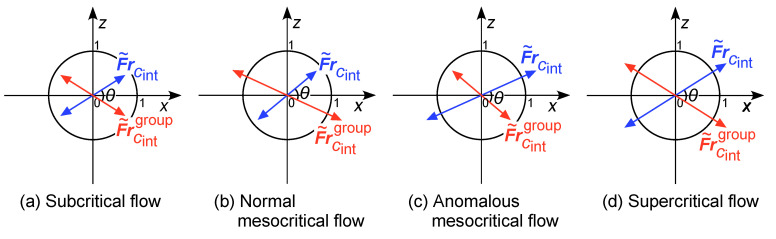
Classification of flow regimes for internal gravity waves of fixed k, based on the component-wise phase and group Froude vectors (50) relative to the unit circle (drawn for $k_{x},k_{z}>0$ and $\bar{u}>\bar{w}>0$, giving the principal branch $\theta \in [0,\frac{\pi }{2}]$, under the constraint ${\tilde{Fr}}_{c_{\mathrm{int}},z}=-{\tilde{Fr}}_{c_{\mathrm{int}},z}^{\mathrm{group}}$).

This is a function of $\bar{u}$ and k, with two supplementary solutions for θ∈[0,π], so the vectors are double-headed. The flow regimes identified by these Froude numbers can therefore be classified by the “clock” vector diagrams shown in Figure 1, based on the magnitude of each Froude vector relative to the unit circle in the [x,z] plane.

In previous studies, various summary definitions are used, including $Fr_{y}=U/N_{0}y$, $Fr_{\ell }=U/N_{0}\ell$, $Fr_{k}=Uk/N_{0}$ or the Long or Russell number $Lo=N_{0}h/U$, where *U* is a summary velocity [m s${}^{-1}$], *ℓ* is a horizontal length scale [m] and *h* is vertical step length scale [m] [101,112,120]. These have been used to distinguish simple subcritical (Fr<1) and supercritical (Fr>1) flow regimes for internal gravity waves. Furthermore, the gradient Richardson number $Ri=N_{0}^{2}/{\left(\partial u/\partial z\right)}^{2}$ can be used to discriminate between buoyancy-dominated (Ri≫1) and shear-dominated (Ri≪1) flows [102].

### 4.8. Inertial Waves

For a fluid on a rotating body such as the Earth, the apparent force (Coriolis effect) created by the non-inertial frame of reference can act as a restoring force, generating wave motion. There are three main categories: (i) *pure inertial waves* with transverse oscillations, dominated by the rotation rate; (ii) *inertia–gravity waves* with elliptical oscillations, due to the action of both rotation and buoyancy, and (iii) large-scale *Rossby* or *planetary waves*, caused by variation of the Coriolis effect with latitude [113,116].

On a planetary surface, the strength of rotation can be represented by the Coriolis parameter f=2Ωsinφ [s${}^{-1}$], where Ω is the angular frequency of rotation [rad s${}^{-1}$] and φ is the latitude [112,115,116,119]. For two-dimensional inertia–gravity waves subject to the Boussinesq approximation, the angular frequency and phase celerity are [101,114,115,117,119]:

(53) $$\begin{array}{cc} \omega^{2}=\frac{\left|\right|k^{*}{\left|\right|}^{2}}{{\left||k|\right|}^{2}} & c_{in-gr}=\frac{\omega k}{{\left||k|\right|}^{2}}=\pm \frac{\left|\right|k^{*}\left|\right|\;k}{{\left||k|\right|}^{3}}, \end{array}$$

where $\left|\right|k^{*}\left|\right|=\sqrt{N_{0}^{2}k_{x}^{2}+f^{2}k_{z}^{2}}$. This has the limits f→0 for pure gravity waves (Equation (48)) and $N_{0}\to 0$ for pure inertial waves. Applying the definition in Equation (48), the group celerity is [117]:

(54) $$\begin{array}{cc} \; & c_{\mathrm{in-gr}}^{\mathrm{group}}=\nabla_{k}\omega =\pm \frac{\left(N_{0}^{2}-f^{2}\right)k_{x}k_{z}Rk}{\left|\right|k^{*}{\left||\;||k|\right|}^{3}}=\pm \frac{\left(N_{0}^{2}-f^{2}\right)k_{x}k_{z}}{\left|\right|k^{*}{\left||\;||k|\right|}^{3}}\;\left[\begin{array}{c} k_{z}\\ -k_{x} \end{array}\right] \end{array}$$

This again gives $c_{\mathrm{in-gr}}\cdot c_{\mathrm{in-gr}}^{\mathrm{group}}=0$ [117], so the phase and group celerities are orthogonal. The component-wise phase and group Froude vectors (50) become:

(55) $$\begin{array}{c} \begin{array}{cc} {\tilde{\Pi }}_{c_{\mathrm{in-gr}}}={\tilde{\mathrm{Fr}}}_{c_{\mathrm{in-gr}}}\; & =\bar{u}⊘c_{\mathrm{in-gr}}=\pm \frac{{\left||k|\right|}^{3}}{\left|\right|k^{*}\left|\right|}\;\bar{u}⊘k=\pm \frac{{\left||k|\right|}^{3}}{\left|\right|k^{*}\left|\right|}\;\left[\begin{array}{c} \bar{u}/k_{x}\\ \bar{w}/k_{z} \end{array}\right]\\ {\tilde{\Pi }}_{c_{\mathrm{in-gr}}}^{\mathrm{group}}={\tilde{\mathrm{Fr}}}_{c_{\mathrm{in-gr}}}^{\mathrm{group}}\; & =\bar{u}⊘c_{\mathrm{in-gr}}^{\mathrm{group}}=\pm \frac{\left|\right|k^{*}{\left||\;||k|\right|}^{3}}{N_{0}^{2}-f^{2}}\;\left[\begin{array}{c} \bar{u}/k_{x}k_{z}^{2}\\ -\bar{w}/k_{x}^{2}k_{z} \end{array}\right], \end{array} \end{array}$$

These are separated by an oblique angle θ. The vector definitions (51) give:

(56) $$\begin{array}{c} \begin{array}{cc} {\hat{\Pi }}_{c_{\mathrm{in-gr}}}\; & ={\hat{\mathrm{Fr}}}_{c_{\mathrm{in-gr}}}=\frac{\bar{u}}{\left|\right|c_{\mathrm{in-gr}}\left|\right|}=\frac{{\left||k|\right|}^{2}\;\bar{u}}{\left|\right|k^{*}\left|\right|}\\ {\hat{\Pi }}_{c_{\mathrm{in-gr}}}^{\mathrm{group}}\; & ={\hat{\mathrm{Fr}}}_{c_{\mathrm{in-gr}}}^{\mathrm{group}}=\frac{\bar{u}}{\left|\right|c_{\mathrm{in-gr}}^{\mathrm{group}}\left|\right|}=\frac{\left|\right|k^{*}{\left||\;||k|\right|}^{2}\;\bar{u}}{|\left(N_{0}^{2}-f^{2}\right)k_{x}k_{z}|} \end{array} \end{array}$$

Equations (55) and (56) again suggest the existence of four information-theoretic flow regimes in flows with inertia-gravity waves, as proposed for internal gravity waves (Section 4.7). These warrant further investigation. The component-wise definitions (55) can again be represented by clock vector diagrams such as those in Figure 1. In both definitions (55) and (56), inertia–gravity systems exhibit a transition between dominance by gravity waves ($N_{0}^{2}>f^{2}$) or inertial waves ($N_{0}^{2}<f^{2}$), producing a discontinuity in the group Froude vectors. On planet Earth, generally $N_{0}^{2}>f^{2}$, so the gravity term will be dominant [119].

In contrast, for three-dimensional Rossby waves on planet Earth with coordinates $x={\left[x,y,z\right]}^{⊤}$ and wavenumber $k={\left[k_{x},k_{y},k_{z}\right]}^{⊤}$ oriented east, north and upwards, respectively, by the conservation of absolute vorticity subject to the Boussinesq approximation and $f=f_{0}+\beta y$ with parameters $f_{0}>0$ [s${}^{-1}$] and β>0 [m${}^{-1}$ s${}^{-1}$], the intrinsic angular frequency and celerity are [112,113,115,117,119]:

(57) $$\begin{array}{c} \omega =-\frac{\beta k_{x}}{\left|\right|k^{†}{\left|\right|}^{2}},\;c_{Ro}=\frac{\omega k}{{\left||k|\right|}^{2}}=-\frac{\beta k_{x}k}{\left|\right|k^{†}{\left|\right|}^{2}{\left||k|\right|}^{2}} \end{array}$$

where $\left|\right|k^{†}\left|\right|=\sqrt{k_{x}^{2}+k_{y}^{2}+\phi_{0}^{2}k_{z}^{2}}$ with $\phi_{0}=f_{0}/N_{0}$. As evident, Rossby waves are single-directional. Since $c_{Ro,x}<0$, Rossby waves migrate westwards relative to the mean flow, while since $f_{0}^{2}<N_{0}^{2}$ or $\phi_{0}\to 0$ on Earth, the vertical component is small [113,117]. From the definition in Equation (48), the group celerity is (c.f., [115,117,119]):

(58) $$\begin{array}{cc} \; & c_{Ro}^{\mathrm{group}}=\nabla_{k}\omega =\frac{\beta }{\left|\right|k^{†}{\left|\right|}^{4}}\;\left[\begin{array}{c} k_{x}^{2}-k_{y}^{2}-\phi_{0}^{2}k_{z}^{2}\\ 2k_{x}k_{y}\\ 2\phi_{0}^{2}k_{x}k_{z} \end{array}\right]=\frac{\beta }{\left|\right|k^{†}{\left|\right|}^{4}}\;\left[\begin{array}{c} 2k_{x}^{2}-\left|\right|k^{†}{\left|\right|}^{2}\\ 2k_{x}k_{y}\\ 2\phi_{0}^{2}k_{x}k_{z} \end{array}\right] \end{array}$$

From the dot product $c_{Ro}\cdot c_{Ro}^{\mathrm{group}}$, the angle ψ between the two celerities is given by:

(59) $$\begin{array}{c} \cos\psi =-\frac{|k_{x}\left|\;|\right|k^{†}{\left|\right|}^{2}}{\left||k|\right|\sqrt{4\phi_{0}^{2}\left(\phi_{0}^{2}-1\right)k_{x}^{2}k_{z}^{2}+\left|\right|k^{†}{\left|\right|}^{4}}} \end{array}$$

In consequence, individual Rossby waves and wave groups are neither orthogonal nor parallel, but meet at an oblique angle (e.g., [117,119]).

The component-wise Froude vectors for flows with Rossby waves of fixed wavenumber and direction k in Equation (50) are:

(60) $$\begin{array}{c} \begin{array}{cc} {\tilde{\Pi }}_{c_{Ro}}={\tilde{\mathrm{Fr}}}_{c_{Ro}}\; & =\bar{u}⊘c_{Ro}=-\frac{\left|\right|k^{†}{\left|\right|}^{2}{\left||k|\right|}^{2}}{\beta }\left[\begin{array}{c} \bar{u}/k_{x}^{2}\\ \bar{v}/k_{x}k_{y}\\ \bar{w}/k_{x}k_{z} \end{array}\right],\\ {\tilde{\Pi }}_{c_{Ro}}^{\mathrm{group}}={\tilde{\mathrm{Fr}}}_{c_{Ro}}^{\mathrm{group}}\; & =\bar{u}⊘c_{Ro}^{\mathrm{group}}=\frac{\left|\right|k^{†}{\left|\right|}^{4}}{\beta }\left[\begin{array}{c} \bar{u}/(2k_{x}^{2}-\left|\right|k^{†}{\left|\right|}^{2})\\ \bar{v}/2k_{x}k_{y}\\ \bar{w}/2\phi_{0}^{2}k_{x}k_{z} \end{array}\right], \end{array} \end{array}$$

again separated by an oblique angle θ. The vector definitions for waves of fixed wavenumber and arbitrary direction ||k|| in Equation (51) give:

(61) $$\begin{array}{c} \begin{array}{cc} {\hat{\Pi }}_{c_{Ro}}\; & ={\hat{\mathrm{Fr}}}_{c_{Ro}}=\frac{\bar{u}}{\left|\right|c_{Ro}\left|\right|}=\frac{\left|\right|k^{†}{\left|\right|}^{2}||k||\;\bar{u}}{\beta |k_{x}|}\\ {\hat{\Pi }}_{c_{Ro}}^{\mathrm{group}}\; & ={\hat{\mathrm{Fr}}}_{c_{Ro}}^{\mathrm{group}}=\frac{\bar{u}}{\left|\right|c_{Ro}^{\mathrm{group}}\left|\right|}=\frac{\left|\right|k^{†}{\left|\right|}^{4}\;\bar{u}}{\beta ||k^{‡}\left|\right|} \end{array} \end{array}$$

where $\left|\right|k^{‡}\left|\right|=\sqrt{{k_{x}}^{4}+2{k_{x}}^{2}{k_{y}}^{2}+{k_{y}}^{4}+2\phi_{0}^{2}\left(2\phi_{0}^{2}-1\right){k_{x}}^{2}{k_{z}}^{2}+2\phi_{0}^{2}{k_{y}}^{2}{k_{z}}^{2}+\phi_{0}^{4}{k_{z}}^{4}}$.

**Figure 2:**
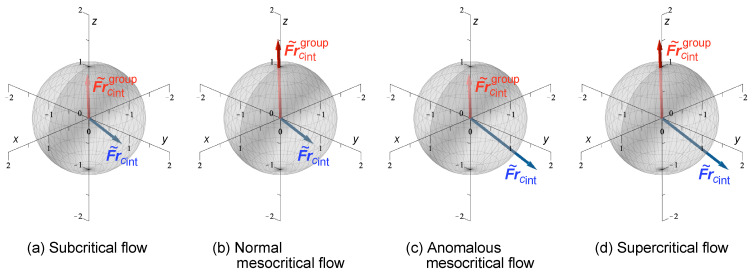
Classification of flow regimes for Rossby waves of fixed wavenumber and direction, based on the component-wise phase and group Froude vectors in Equation (60) relative to the unit sphere (drawn for $k_{x},k_{y},k_{z}>0$ and $\bar{u}>\bar{w}>0>\bar{v}$ with $f_{0}\ll N_{0}$).

Equations (60) and (61) again suggest the existence of four information-theoretic flow regimes in flows with Rossby waves, as proposed for internal gravity and inertia-gravity waves (Section 4.7 and Section 4.8). These warrant further investigation. The component-wise definitions (60) can be classified by the “gyroscope” three-dimensional vector diagrams shown in Figure 2, based on the magnitude of each Froude vector relative to the unit sphere.

The literature provides a simpler treatment of inertia–gravity systems, applying information-theoretic similarity (19) based on distinct stratification and rotational celerity scales $c_{\mathrm{strat}}$ and $c_{\mathrm{rot}}$ to give the Froude and Rossby numbers, respectively [112]:

(62) $$\begin{array}{cccc} \Pi_{c_{\mathrm{strat}}} & =\frac{U}{c_{\mathrm{strat}}}∼Fr_{N_{0}}=\frac{U}{N_{0}h} & & \to \;Fr_{h}=\frac{U}{\sqrt{gh}}, \end{array}$$

(63) $$\begin{array}{cccc} \Pi_{c_{\mathrm{rot}}} & =\frac{U}{c_{\mathrm{rot}}}∼Ro_{f}=\frac{U}{f\ell } & & \to \;Ro_{\Omega }=\frac{U}{\Omega \ell } \end{array}$$

where *h* and *ℓ* are vertical and horizontal length scales [m]. These have been used as discriminators, respectively, of inertial transport relative to wave motion governed by stratification or rotation [112]. The Burger number $Bu=Ro^{2}/Fr^{2}={\left(N_{0}h/f\ell \right)}^{2}$ directly ranks the last two effects.

Many other formulations have been provided for inertial waves under different conditions, including for non-Boussinesq fluids, anelastic internal waves, acoustic and inertia-gravity waves in combination, bounded waves, rotating shallow water waves, baroclinic waves, thermocline effects, Kelvin waves, equatorial waves, lee and solitary waves, solar and lunar tidal waves, and a large assortment of seasonal, oceanographic and climatic oscillations [101,112,113,114,115,116,117,119,121].

### 4.9. Electromagnetic Waves

#### 4.9.1. Dimensionless Groups and Electromagnetic Flow Regimes

Electromagnetic waves consist of synchronized transverse oscillations of electric and magnetic fields, quantized in the form of photons. Applying information-theoretic similarity (19), the speed *u* [m s${}^{-1}$] of an object (such as a subatomic particle) relative to the speed of light in a vacuum $c_{0}$ [m s${}^{-1}$]—both measured in a common inertial frame of reference—defines the dimensionless group:

(64) $$\begin{array}{c} \Pi_{c_{0}}=\frac{u}{c_{0}} \end{array}$$

This can be used to discriminate between two flow regimes:

1. *Subluminal flow* ($\Pi_{c_{0}}<1$), subject to the influence of electromagnetic signals; and
2. *Superluminal flow* ($\Pi_{c_{0}}>1$), which cannot be influenced by electromagnetic signals.

However, from special relativity, superluminal phenomena are not permissible in the universe. Instead, a material object with $u\to c_{0}$ will undergo time dilation and length contraction in accordance with the Lorentz transformations, while the simultaneity of events for different observers is broken [122]. Despite considerable interest in this topic (e.g., [123,124,125,126,127,128,129]), a technology to achieve $\Pi_{c_{0}}>1$ remains a dream of science fiction writers and movie producers.

Electromagnetic waves transmitted through a physical material exhibit wave refraction, leading to the phase celerity $c\neq c_{0}$, and wave dispersion, creating wave packets with the group celerity $c^{\mathrm{group}}\neq c$. These are given by [62,128,130,131,132]:

(65) $$\begin{array}{c} \begin{array}{c} \omega =\frac{c_{0}k}{n},\;c=\frac{\omega }{k}=\frac{c_{0}}{n},\;c^{\mathrm{group}}=\frac{d\omega }{dk}=\frac{c}{1+\frac{\omega }{n}\frac{dn}{d\omega }}=c\left(1-\frac{k}{n}\frac{dn}{dk}\right) \end{array} \end{array}$$

where ω is the wave frequency [s${}^{-1}$] and *n* is the refractive index [-]. In usual circumstances in a non-attenuating medium, the group celerity corresponds to the *signal velocity* of the electromagnetic wave packet [62].

Equation (65) gives the information-theoretic dimensionless groups:

(66) $$\begin{array}{c} \Pi_{c}=\frac{u}{c}=\frac{nu}{c_{0}}=n\Pi_{c_{0}},\;\Pi_{c}^{\mathrm{group}}=\frac{u}{c^{\mathrm{group}}}=\frac{u(1+\frac{\omega }{n}\frac{dn}{d\omega })}{c}=\frac{u}{c\left(1-\frac{k}{n}\;\frac{dn}{dk}\right)} \end{array}$$

Common materials exhibit normal refraction n>1, hence $c<c_{0}$ and $\Pi_{c}>\Pi_{c_{0}}$, and normal wave dispersion dn/dk>0 and $c^{\mathrm{group}}<c$, hence $\Pi_{c}^{\mathrm{group}}>\Pi_{c}$. It then becomes permissible to achieve $\Pi_{c}^{\mathrm{group}}>1$ and at high particle velocities $\Pi_{c}>1$. Indeed, the interference of waves of different frequencies can produce stationary wave packets with $c^{\mathrm{group}}=0$, giving $\Pi_{c}^{\mathrm{group}}=\infty$ [133]. Extending the previous analyses, the three different vacuum, phase and group celerities for electromagnetic waves suggest the existence of four regimes for the motion of particles in a normal dispersive medium:

1. *Subluminal flow* ($\Pi_{c_{0}}<\Pi_{c}<\Pi_{c}^{\mathrm{group}}<1$), subject to the influence of electromagnetic waves and wave groups;
2. *Normal mesoluminal flow* ($\Pi_{c_{0}}<\Pi_{c}<1<\Pi_{c}^{\mathrm{group}}$), influenced by individual electromagnetic waves but not wave groups;
3. *Transluminal flow* ($\Pi_{c_{0}}<1<\Pi_{c}<\Pi_{c}^{\mathrm{group}}$), which cannot be influenced by electromagnetic waves or wave groups, but which is permitted under special relativity; and
4. *Superluminal flow* ($1<\Pi_{c_{0}}<\Pi_{c}<\Pi_{c}^{\mathrm{group}}$), precluded by special relativity.

There is some confusion in the literature over the definition of superluminal flow, applied by some authors to u>c as well as $u>c_{0}$. The above definitions provide a more precise terminology. The transitions can be described as *vacuum* or *universal luminal flow* for $\Pi_{c_{0}}=1$, *luminal flow* for $\Pi_{c}=1$, and *group luminal flow* for $\Pi_{c}^{\mathrm{group}}=1$.

In contrast, some materials exhibit anomalous refraction n<1, hence $c>c_{0}$, and/or anomalous dispersion dn/dk<0 and $c^{\mathrm{group}}>c$, even to the extent that $c^{\mathrm{group}}>c_{0}$ [128,132,134,135]. This creates the possibility of *anomalous mesoluminal flow* ($\Pi_{c}^{\mathrm{group}}<1<\Pi_{c}$), influenced by electromagnetic wave groups but not individual waves. For the other flow regimes, $\Pi_{c}$ and $\Pi_{c}^{\mathrm{group}}$ are interchanged. Concerning the enhanced celerities, while individual waves with $c>c_{0}$ can arise for certain frequency bands in some materials, and wave packets can be constructed from individual waves such that $c^{\mathrm{group}}>c_{0}$, it has been shown that these cannot be used to transmit superluminal signals, since the signal velocity—now equal to the *wave front celerity* or *edge celerity* of the wave motion—cannot exceed the vacuum celerity $c_{0}$ [62,128,130,131,132].

Transluminal flow is known, revealed by the emission of Vavilov–Cherenkov electromagnetic radiation within the shock front created by transluminal charged particles in a dielectric medium [136,137,138]. This produces the bluish glow around nuclear fuel elements stored underwater. Apart from this, the physical manifestations of the postulated mesoluminal and anomalous mesoluminal flow regimes for particle flows influenced by electromagnetic waves—and the roles of the phase, group and edge celerities—warrant further study.

A more complicated formulation of electromagnetic wave opacity can be expressed using a complex refractive index $n^{*}=n+ı\kappa$, where $ı=\sqrt{-1}$ and κ∈R is the absorption or extinction coefficient [62,128,132,139,140]. This definition allows for negative or complex celerities. By information-theoretic similarity (19), these will give corresponding negative or complex dimensionless groups $\Pi_{c}$ and $\Pi_{c}^{\mathrm{group}}$, leading to a complicated set of information-theoretic transmission and absorption regimes defined over the complex domain. These warrant further study.

#### 4.9.2. Radiative Entropy Flux and Entropic Groups

The transport of entropy by electromagnetic radiation (or subatomic particles) and its influence on the entropy production is an important phenomenon, often omitted from standard analyses of heat transfer or radiative processes. The *radiative energy flux*, *energy irradiance* or *energy fluence rate* $j_{E,\mathrm{rad}}$ [W m${}^{-2}$] and the *radiative entropy flux*, *entropy irradiance* or *entropy fluence rate* $j_{S,\mathrm{rad}}$ [W K${}^{-1}$ m${}^{-2}$] of electromagnetic radiation striking an infinitesimal area with unit normal n are given, respectively, by [65,141,142,143,144,145,146]:

(67) $$\begin{array}{cc} j_{E,\mathrm{rad}} & =n\;\int_{0}^{\infty }\underset{\Omega }{\;\int \int \;}I_{\mathrm{rad}}\;m\cdot n\;d\Omega \;d\omega \end{array}$$

(68) $$\begin{array}{cc} j_{S,\mathrm{rad}} & =n\;\int_{0}^{\infty }\underset{\Omega }{\;\int \int \;}L_{\mathrm{rad}}\;m\cdot n\;d\Omega \;d\omega \end{array}$$

where $I_{\mathrm{rad}}$ is the specific energy intensity or energy radiance [W m${}^{-2}$ s sr${}^{-1}$], the radiation energy per unit frequency travelling through an infinitesimal area of unit normal m and infinitesimal solid angle per unit time, $L_{\mathrm{rad}}$ is the analogous specific entropy intensity or entropy radiance [W K${}^{-1}$ m${}^{-2}$ s sr${}^{-1}$] and Ω is the solid angle [sr]. As evident from Equation (68), the entropy irradiance is a property of the radiation itself, distinct from the entropy produced by its conversion to heat. Thus in the presence of electromagnetic radiation, in addition to the non-radiative (material) component in Equation (4), there is a separate radiative component to the local entropy production [142,143,144,145,147]:

(69) $$\begin{array}{cc} {\hat{\dot{\sigma }}}_{\mathrm{rad}}\; & =\frac{\partial }{\partial t}{\hat{S}}_{\mathrm{rad}}+\nabla \cdot j_{S,\mathrm{rad}} \end{array}$$

where ${\hat{S}}_{\mathrm{rad}}$ is the radiation entropy concentration [J K${}^{-1}$ m${}^{-3}$]. The Clausius heating term $j_{E,\mathrm{rad}}/T$ due to the radiative energy flux must also be added to the thermodynamic entropy flux $j_{S}$ in Equation (4) [142,143,144].

For unpolarized bosons such as photons, the specific entropy intensity is given by [142,143,144,145,148]:

(70) $$\begin{array}{cc} L_{\mathrm{rad}} & =\frac{2k_{B}\omega^{2}}{c^{2}}\left[\left(\frac{c^{2}I_{\mathrm{rad}}}{2h\omega^{3}}+1\right)\ln\left(\frac{c^{2}I_{\mathrm{rad}}}{2h\omega^{3}}+1\right)-\frac{c^{2}I_{\mathrm{rad}}}{2h\omega^{3}}\ln\frac{c^{2}I_{\mathrm{rad}}}{2h\omega^{3}}\right] \end{array}$$

where $k_{B}$ is Boltzmann’s constant [J K${}^{-1}$] and *h* is the Planck constant [J s]. Other relations are available for polarized radiation [29,141] or fermions [149]. For unpolarized bosons emitted by a black body of radiative temperature $T_{\mathrm{rad}}$, $I_{\mathrm{rad}}$ is given by Planck’s law [29,141,144,148,150]:

(71) $$\begin{array}{cc} I_{\mathrm{rad}} & =\frac{2h\omega^{3}}{c^{2}}\frac{1}{\exp(h\omega /k_{B}T_{\mathrm{rad}})-1} \end{array}$$

Substituting Equations (70) and (71) into Equations (67) and (68) and integrating over the frequency and a hemisphere Ω∈[0,2π], using m·n=cosϑ and dΩ=sinϑdϑdφ, where ϑ and φ are respectively the colatitude and longitude in spherical coordinates, gives:

(72) $$\begin{array}{c} \begin{array}{cc} j_{E,\mathrm{rad}} & =\frac{2\pi^{5}k_{B}^{4}T_{\mathrm{rad}}^{4}}{15c^{2}h^{3}}n=\tilde{\sigma }T_{\mathrm{rad}}^{4}\;n,\\ j_{S,\mathrm{rad}} & =\frac{8\pi^{5}k_{B}^{4}T_{\mathrm{rad}}^{3}}{45c^{2}h^{3}}=\frac{4}{3}\tilde{\sigma }T_{\mathrm{rad}}^{3}\;n \end{array} \end{array}$$

where $\tilde{\sigma }=2\pi^{5}k_{B}^{4}/15c^{2}h^{3}$ is the Stefan–Boltzmann constant [142,150]. For emissions by or interactions with a surface, Equation (72) is multiplied by a fractional emissivity $\tilde{\epsilon }$, absorptivity $\tilde{\alpha }$, reflectivity $\tilde{\rho }$ or transmissivity $\tilde{\tau }$ [-], with $\tilde{\epsilon }=\tilde{\alpha }$ and $\tilde{\alpha }+\tilde{\rho }+\tilde{\tau }=1$ [146,151].

We can now construct entropic dimensionless groups from the entropy fluxes for radiation (Equation (72)), fluid transport ρsu, or the diffusion of heat, chemical species or charged particles (see [20]):

(73) $$\begin{array}{c} \begin{array}{cccc} {\hat{\Pi }}_{j_{S,\mathrm{rad}}/j_{E,\mathrm{rad}}}\; & =\frac{\left|\right|j_{S,\mathrm{rad}}\left|\right|}{\left|\right|j_{E,\mathrm{rad}}/T\left|\right|}=\frac{4T}{3T_{\mathrm{rad}}},\\ {\hat{\Pi }}_{j_{S,\mathrm{rad}}/j_{S,f}}\; & =\frac{\left|\right|j_{S,\mathrm{rad}}\left|\right|}{\left|\right|j_{S,f}\left|\right|}∼\frac{\tilde{\sigma }T_{\mathrm{rad}}^{3}}{\rho s||u||},\\ {\hat{\Pi }}_{j_{S,\mathrm{rad}}/j_{S,\alpha }}\; & =\frac{\left|\right|j_{S,\mathrm{rad}}\left|\right|}{\left|\right|j_{S,\alpha }\left|\right|}∼\frac{\tilde{\sigma }T\;T_{\mathrm{rad}}^{3}}{\left|\right|j_{Q}\left|\right|}∼\frac{\tilde{\sigma }T_{\mathrm{rad}}^{3}}{\alpha \rho c_{p}T\;||\nabla T^{-1}\left|\right|}\; & \to Sk & =\frac{\tilde{\sigma }\ell \;T_{\mathrm{rad}}^{3}}{\alpha \rho c_{p}}=\frac{\tilde{\sigma }\ell \;T_{\mathrm{rad}}^{3}}{k},\\ {\hat{\Pi }}_{j_{S,\mathrm{rad}}/j_{S,D_{c}}}\; & =\frac{\left|\right|j_{S,\mathrm{rad}}\left|\right|}{\left|\right|j_{S,D_{c}}\left|\right|}∼\frac{\tilde{\sigma }T\;T_{\mathrm{rad}}^{3}}{\mu_{c}\;||j_{c}\left|\right|}∼\frac{\tilde{\sigma }RT\;T_{\mathrm{rad}}^{3}}{D_{c}\rho m_{c}\mu_{c}\;||\nabla \frac{\mu_{c}}{T}\left|\right|},\\ {\hat{\Pi }}_{j_{S,\mathrm{rad}}/j_{S,D_{k}}}\; & =\frac{\left|\right|j_{S,\mathrm{rad}}\left|\right|}{\left|\right|j_{S,D_{k}}\left|\right|}∼\frac{\tilde{\sigma }T\;T_{\mathrm{rad}}^{3}}{\Phi \;||i_{k}\left|\right|}∼\frac{\tilde{\sigma }RT^{2}\;T_{\mathrm{rad}}^{3}}{D_{k}z_{k}^{2}F^{2}C_{k}\Phi \;||\nabla \Phi ||}, \end{array} \end{array}$$

in which, for the diffusion of heat, $j_{Q}$ is the heat flux [J m${}^{-2}$ s${}^{-1}$], α is the thermal diffusion coefficient [m${}^{2}$ s${}^{-1}$] and *ℓ* is a length scale [m]; for the diffusion of chemical species *c*, $j_{c}$ is the molar flux [(mol species) m${}^{-2}$ s${}^{-1}$], $\mu_{c}$ is the chemical potential [J (mol species)${}^{-1}$], $D_{c}$ is the diffusion coefficient [m${}^{2}$ s${}^{-1}$] and $m_{c}$ is the molality *c* [(mol species) kg${}^{-1}$], while for the diffusion of charged species *k*, $i_{k}$ is the charge flux [A m${}^{-2}$], Φ is the electrical potential [V = J C${}^{-1}$], $D_{k}$ is the diffusion coefficient [m${}^{2}$ s${}^{-1}$], $C_{k}$ is the molar concentration [(mol species) m${}^{-3}$], *F* is the Faraday constant [C (mol charge)${}^{-1}$] and $z_{k}$ is the charge number (valency) [(mol charge) (mol species)${}^{-1}$]. The first group shows that the energy and entropy radiances at an equilibrium temperature of $T=T_{\mathrm{rad}}$ carry $\frac{3}{7}$ and $\frac{4}{7}$ of the radiation entropy, respectively [142]. The second group, comparing radiative transfer and fluid flow, is related to the reciprocal of the Boltzmann or Thring number $Th=\rho c_{p}U/\tilde{\epsilon }\tilde{\sigma }T_{\mathrm{rad}}^{3}$ with fluid velocity *U* [m s${}^{-1}$] [152]. The third group, comparing radiative transfer and heat conduction, reduces to the Stefan or Stark number Sk for heat transfer by radiation relative to conduction [152]. The final two groups, which respectively compare radiative transfer to chemical or charge diffusion, have applications to photochemical and photovoltaic processes; these are less readily interpreted by dynamic similarity.

Additional entropic groups for radiation can be defined using the entropy fluxes for thermodynamic cross-phenomena, or directly from the entropy production terms for radiation, diffusion and chemical reaction processes (see [20]). The group $h_{Q}/\tilde{\sigma }T_{\mathrm{rad}}^{3}$, where $h_{Q}$ is the heat transfer coefficient [J K${}^{-1}$ m${}^{-2}$ s${}^{-1}$], has been defined to compare heat fluxes by convection and radiation [153].

## 5. Conclusions

Part I of this study [20] proposes a new interpretation for a large class of dimensionless groups based on the principle of *entropic similarity*, involving ratios of (i) entropy production terms; (ii) entropy flow rates or fluxes; or (iii) information flow rates or fluxes. Since all processes involving work against friction, dissipation, diffusion, dispersion, mixing, separation, chemical reaction, gain of information or other irreversible changes are driven by (or must overcome) the second law of thermodynamics, it is appropriate to analyze these processes directly in terms of competing entropy-producing and -transporting phenomena and the dominant entropic regime, rather than indirectly in terms of their associated forces. These definitions are used in Part I to derive entropic groups for a number of entropy-producing and -transporting phenomena, including diffusion and chemical reaction processes, dispersion mechanisms and diffusion in the universe [20].

In this Part II, after a recap of fundamental concepts (Section 3), a number of wave phenomena are examined in detail (Section 4), including acoustic waves, blast waves, pressure waves, capillary waves, surface and internal gravity waves, inertial waves and electromagnetic waves. For each wave type, the information-theoretic definition of similarity and its role as a discriminator between different entropic flow regimes is examined in detail. Detailed entropic analyses of several flow systems are also presented, including the formation of sharp transitions, the frictional behavior of different flow regimes, and additional mechanisms for entropy transport. Comparing these analyses with those obtained by traditional methods, we can draw several conclusions:

1. The information-theoretic definition of similarity in Equations (16) and (17) and (19) and (20) provides the foundation for a number of dimensionless groups as ratios of the fluid or particle velocity to the prevalent signal velocity. Globally Π<1 or locally $\hat{\Pi }<1$, a signal (manifested by a wave) can to be transported upstream, thereby influencing the flow, while for Π>1 or $\hat{\Pi }>1$, this is not possible, leading to two distinct downstream- and upstream-controlled information-theoretic flow regimes. Dimensionless groups in this category include:
  (a) Mach numbers for acoustic waves (21) and blast waves (30), defining the transition from subsonic to supersonic flow. A Mach number in the form of an Euler number also arises for pressure waves (water hammer) (32).
  (b) Froude numbers for surface gravity waves (34)–(38), surface gravity–capillary waves (44)–(46), internal gravity waves (50) and (51) and inertial waves (55) and (56), defining the transition from subcritical to supercritical flow.
  (c) A Rossby number for rotational inertial waves (63), defining the transition from inertia-dominated to rotation-dominated flows.
  (d) A new set of dimensionless groups for flows of particles influenced by electromagnetic waves (64)–(66), defining the transition between subluminal and superluminal flow.
  The above named groups are traditionally interpreted by dynamic similarity: the Mach number as the ratio of inertial and elastic forces, the Euler number as the ratio of pressure and inertial forces, the Froude number as the ratio of inertial and gravity forces and the Rossby number as the ratio of inertial and rotational forces.
2. For fluid flows influenced by dispersive waves, there exist two distinct celerities for individual waves *c* and wave groups $c^{\mathrm{group}}$, with corresponding information-theoretic groups $\Pi_{c}$ and $\Pi_{c}^{\mathrm{group}}$. It is postulated that these allow for the existence of multiple information-theoretic flow regimes, as follows:
  (a) *Subsonic* or *subcritical* flow ($\{\Pi_{c},\Pi_{c}^{\mathrm{group}}\}<1$), influenced by individual waves and wave groups;
  (b) *Normal mesosonic* or *mesocritical* flow ($\Pi_{c}<1<\Pi_{c}^{\mathrm{group}}$) for flows with normal wave dispersion $c^{\mathrm{group}}<c$, influenced by individual waves but not wave groups;
  (c) *Anomalous mesosonic* or *mesocritical* flow ($\Pi_{c}^{\mathrm{group}}<1<\Pi_{c}$) for flows with anomalous wave dispersion $c^{\mathrm{group}}>c$, influenced by wave groups but not individual waves; and
  (d) *Supersonic* or *supercritical* flow ($1<\{\Pi_{c},\Pi_{c}^{\mathrm{group}}\}$), not influenced by wave motion.
  Wave dispersion does not occur in open channel flows (Section 4.5.1), but arises in most other flows with wave motion, including for some acoustic waves (Section 4.1), deepwater to transitional surface gravity waves (Section 4.5.1), surface gravity–capillary waves (Section 4.6), internal gravity waves (Section 4.7) and inertial waves (Section 4.8). The physical manifestations of the postulated normal and anomalous *meso*- flow regimes—including the observable flow transitions and frictional effects—are not known and warrant further experimental and theoretical investigation.
3. For flows of particles influenced by dispersive electromagnetic waves, there exist three distinct celerities: the vacuum celerity $c_{0}$, the individual wave celerity *c* and the group celerity $c^{\mathrm{group}}$, with corresponding information-theoretic groups $\Pi_{c_{0}}$, $\Pi_{c}$ and $\Pi_{c}^{\mathrm{group}}$. It is postulated that these allow for the existence of multiple information-theoretic flow regimes, as follows:
  (a) *Subluminal flow* ($\Pi_{c_{0}}<\left\{\Pi_{c},\Pi_{c}^{\mathrm{group}}\right\}<1$), influenced by individual waves and wave groups;
  (b) *Normal mesoluminal flow* ($\Pi_{c_{0}}<\Pi_{c}<1<\Pi_{c}^{\mathrm{group}}$) for flows with normal wave dispersion $c^{\mathrm{group}}<c$, influenced by individual waves but not wave groups;
  (c) *Anomalous mesoluminal flow* ($\Pi_{c_{0}}<\Pi_{c}^{\mathrm{group}}<1<\Pi_{c}$) for flows with anomalous wave dispersion $c^{\mathrm{group}}>c$, influenced by wave groups but not individual waves;
  (d) *Transluminal flow* ($\Pi_{c_{0}}<1<\left\{\Pi_{c},\Pi_{c}^{\mathrm{group}}\right\}$), which cannot be influenced by electromagnetic waves or wave groups, but which is permitted under special relativity; and
  (e) *Superluminal flow* ($1<\Pi_{c_{0}}<\left\{\Pi_{c},\Pi_{c}^{\mathrm{group}}\right\}$), precluded by special relativity.
  Transluminal flow is known, revealed by Vavilov–Cherenkov radiation from a nuclear source in a dielectric medium [136,137,138]. The physical manifestations of the postulated normal and anomalous mesoluminal flow regimes—or possible transport and attenuation regimes defined over the complex domain—are not known, and warrant further study.
4. In flows with multidimensional waves, wave dispersion causes the individual and group wave celerities to act in different directions: orthogonal for internal gravity waves, pure inertial waves and inertia–gravity waves, and at an oblique angle for Rossby waves (Section 4.7 and Section 4.8). These respectively give component-wise vector Froude numbers (50), (55) and (60) for waves of fixed wavenumber and direction, and vector Froude numbers (51), (56) and (61) for waves of fixed wavenumber and arbitrary direction. The first set produce complicated patterns of directional information-theoretic flow regimes, which can be represented by the “clock” or “gyroscopic” vector plots shown in Figure 1 and Figure 2, respectively, for two- or three-dimensional flows.
5. The entropic perspective is also shown to provide a more natural interpretation of sharp transitions between flow regimes and their frictional behavior, including the occurrence of shock waves (Equation (25)) and frictional choking (Equation (26)) in compressible flows, and the occurrence of hydraulic jumps (Equation (40)) and different surface flow profiles (Equation (42)) in open channel flows.
6. The entropic perspective also provides a framework for the analysis of entropy transport by radiation, giving several new and existing dimensionless groups for the competition between radiative, energetic and diffusion processes (Equation (73)).

To conclude, it is shown that the information-theoretic definition of entropic similarity—available in scalar and vector forms in Equations (16), (17), (19) and (20)—enables the derivation of new dimensionless groups beyond those accessible by geometric, kinematic and dynamic similarity. These reveal the existence of different information-theoretic flow regimes, including the possibility of more than two flow regimes in systems with wave dispersion. These significantly expand the scope of similarity and dimensional arguments for the analysis of flow systems with wave propagation.

Finally, while this and the preceding study [20] examine a number of important entropic phenomena in mass, momentum, energy and charge transfer processes, chemical reactions, dispersion processes and wave propagation relevant to fluid flow systems, they are not claimed to be complete. Many other important processes have not been examined from an entropic similarity perspective, including mixing and separation unit operations in chemical and environmental engineering [154,155], radioactive decay and nuclear processes [156], plasma waves and magnetohydrodynamics [157,158,159], gravitation [160], hydraulic and hydrological systems [161,162], biological growth, evolutionary and planetary processes [34,163,164], transport systems [165,166] and economic systems and industrial ecology [167]. Further research is required on the derivation of entropic dimensionless groups to represent these and many other natural, engineered and human phenomena.

## Figures and Tables

**Figure A1:**
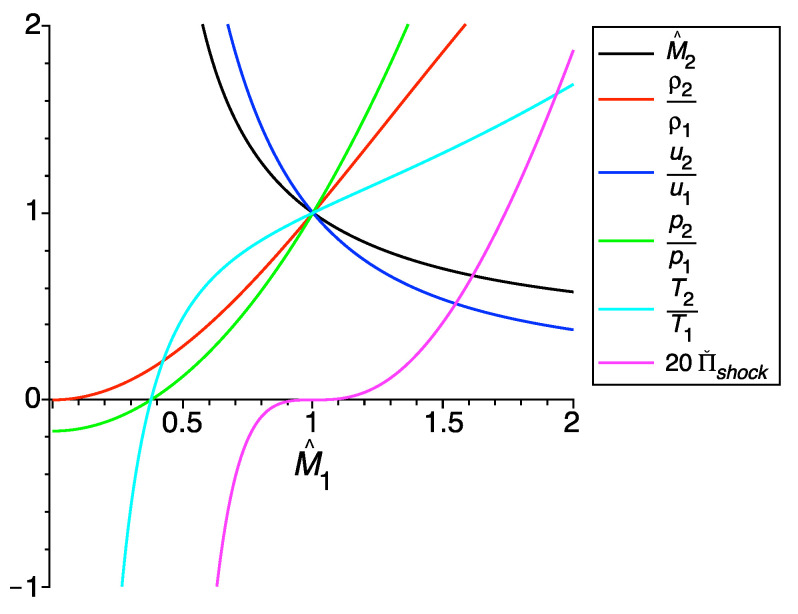
Dimensionless relations (A1) and (25) across a normal shock wave in an inviscid, adiabatic, one-dimensional steady-state flow of compressible dry air.

**Figure A2:**
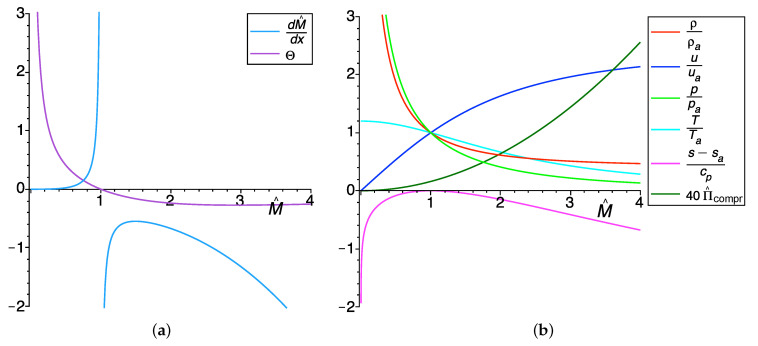
Relations for adiabatic frictional one-dimensional steady-state flow of compressible dry air in a pipe, as functions of $\hat{M}$: (**a**) $d\hat{M}/dx$ (A3) and Θ (26); and (**b**) fluid property ratios (A7) and (A8) and the dimensionless entropy production (26). Note the distinction between the relative specific entropy and the rate of entropy production.

**Figure A3:**
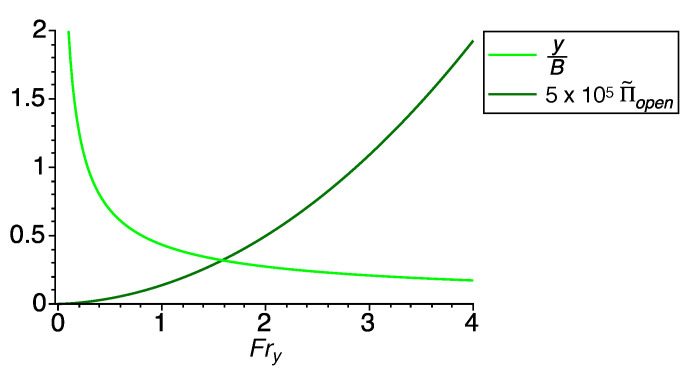
Dimensionless water depth y/B and dimensionless entropy production ${\tilde{\Pi }}_{\mathrm{open}}$ for the example of gradually-varied open channel flow of water at steady state.

**Figure A4:**
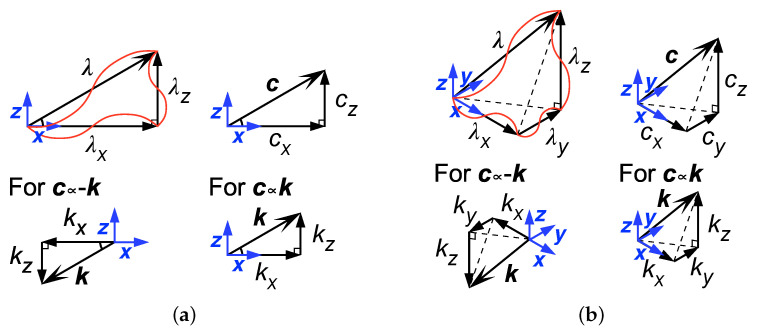
Schematic diagrams of the vector wavelength, phase celerity and wavenumber for (**a**) two-dimensional and (**b**) three-dimensional waves, showing their resolution into components.

**Figure A5:**
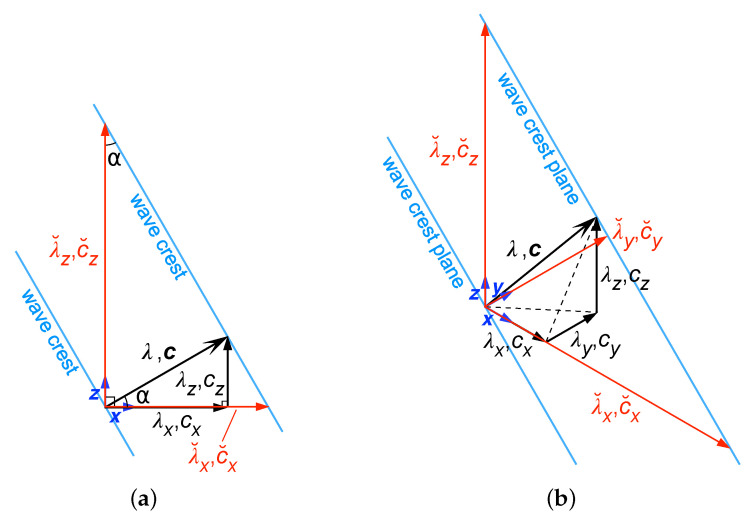
Schematic diagrams of the vector and chord definitions of the wavelength and celerity components, for (**a**) two-dimensional and (**b**) three-dimensional waves.

## Data Availability

Data are contained within the article.

## Appendix A. Shock Waves in Compressible Flow

For the inviscid, adiabatic, one-dimensional steady-state flow of a compressible fluid, by the conservation of fluid mass, momentum and energy in an ideal gas, and the definition of local Mach number (21), the following relations can be derived for fluid properties on the inflow and outflow sides of a normal shock [11,59,69,70,71,72]:

(A1) $$\begin{array}{c} \begin{array}{cc} \frac{\rho_{2}}{\rho_{1}} & =\frac{u_{1}}{u_{2}}=\frac{\left(\gamma +1\right){\hat{M}}_{1}^{2}}{2+\left(\gamma -1\right){\hat{M}}_{1}^{2}}\\ \frac{p_{2}}{p_{1}} & =1+\frac{2\gamma ({\hat{M}}_{1}^{2}-1)}{\gamma +1}\\ \frac{T_{2}}{T_{1}} & =\left(1+\frac{2\gamma ({\hat{M}}_{1}^{2}-1)}{\gamma +1}\right)\frac{2+\left(\gamma -1\right){\hat{M}}_{1}^{2}}{\left(\gamma +1\right){\hat{M}}_{1}^{2}} \end{array} \end{array}$$

The relation for ${\hat{M}}_{2}$ in (25) is also obtained. Furthermore, the Gibbs equation in specific form is dh=Tds+υdp, where $dh=c_{P}dT$ is the change in specific enthalpy [J kg${}^{-1}$] and υ=1/ρ is the specific volume [m${}^{3}$ kg${}^{-1}$]. Integration gives the change in specific entropy:

(A2) $$\begin{array}{cc} s_{2}-s_{1} & =c_{p}\ln\;\left(\frac{T_{2}}{T_{1}}\right)-R^{*}\ln\;\left(\frac{p_{2}}{p_{1}}\right) \end{array}$$

Substituting for the temperature and pressure ratios (A1) and $R^{*}=c_{p}\left(\gamma -1\right)/\gamma$ into (A2) and rescaling by $c_{p}$ gives the last two parts of (25) [11,59,69,70,71,72]. Substitution into the entropy production per unit area (24), assuming continuity $\rho_{2}u_{2}=\rho_{1}u_{1}$, then gives the first two parts of (25). Equation (25) will change for different assumptions, e.g., transitions involving changes in flow rate or heat transfer, or for an oblique shock wave (Section 4.1.2).

For a typical dry air with γ=1.4, $c_{p}=1003$ J K${}^{-1}$ kg${}^{-1}$ and $R^{*}=286.57$ J K${}^{-1}$ kg${}^{-1}$ [11,12], plots of the dimensionless relations in (A1) and (25) as functions of ${\hat{M}}_{1}$ are illustrated in Figure A1. As evident, the dimensionless entropy production ${\overset{˘}{\Pi }}_{\mathrm{shock}}$ is positive for ${\hat{M}}_{1}>1$ and negative for ${\hat{M}}_{1}<1$, with a vanishing entropy production in the limit ${\hat{M}}_{1}\to 1^{\pm }$. These plots confirm that for dry air, the second law of thermodynamics requires that a normal shock can only occur for ${\hat{M}}_{1}>1$.

## Appendix B. Frictional Compressible Flow

Consider frictional one-dimensional steady-state flow of a compressible fluid in a cylindrical pipe of length *L*, with the fluid properties expressed as functions of position 0≤x≤L. From the conservation of fluid mass, momentum and energy in an ideal gas with local Mach number $\hat{M}\left(x\right)$ (21), assuming adiabatic flow dq(x)/dt=0, where q(x) represents the “heat energy concentration” [J kg${}^{-1}$], after some effort, the following differential equations can be obtained [10,17]:

(A3) $$\begin{array}{cc} \frac{d\hat{M}\left(x\right)}{dx} & =-\frac{f\gamma \hat{M}{\left(x\right)}^{3}\left[\left(\gamma -1\right)\hat{M}{\left(x\right)}^{2}+2\right]}{4d_{H}\left(\hat{M}{\left(x\right)}^{2}-1\right)} \end{array}$$

(A4) $$\begin{array}{cc} \frac{1}{\rho (x)}\;\frac{d\rho (x)}{dx}=-\frac{1}{u(x)}\;\frac{du(x)}{dx}\; & =-\frac{2}{\hat{M}\left(x\right)\left[\left(\gamma -1\right)\hat{M}{\left(x\right)}^{2}+2\right]}\;\frac{d\hat{M}\left(x\right)}{dx} \end{array}$$

(A5) $$\begin{array}{cc} \frac{1}{p(x)}\;\frac{dp(x)}{dx} & =-\frac{2\left[\left(\gamma -1\right)\hat{M}{\left(x\right)}^{2}+1\right]}{\hat{M}\left(x\right)\left[\left(\gamma -1\right)\hat{M}{\left(x\right)}^{2}+2\right]}\;\frac{d\hat{M}\left(x\right)}{dx} \end{array}$$

(A6) $$\begin{array}{cc} \frac{1}{T(x)}\;\frac{dT(x)}{dx} & =-\frac{2\hat{M}\left(x\right)\left(\gamma -1\right)}{\left(\gamma -1\right)\hat{M}{\left(x\right)}^{2}+2}\;\frac{d\hat{M}\left(x\right)}{dx} \end{array}$$

The fluid properties (A4)–(A6) are usually expressed in differential form as functions of *f* [11,17,69]. Integration of (A3)–(A6) with the sonic boundary condition $\hat{M}\left(L^{*}\right)=1$ then yields (27) and the relations [10,11,17,69,71]:

(A7) $$\begin{array}{c} \begin{array}{cc} \frac{\rho (x)}{\rho_{a}}=\frac{a}{u(x)} & =\frac{1}{\hat{M}\left(x\right)}\;\sqrt{\frac{\left(\gamma -1\right)\hat{M}{\left(x\right)}^{2}+2}{1+\gamma }}\\ \frac{p(x)}{p_{a}} & =\frac{1}{\hat{M}\left(x\right)}\;\sqrt{\frac{1+\gamma }{\left(\gamma -1\right)\hat{M}{\left(x\right)}^{2}+2}}\\ \frac{T(x)}{T_{a}} & =\frac{1+\gamma }{\left(\gamma -1\right)\hat{M}{\left(x\right)}^{2}+2} \end{array} \end{array}$$

where subscript *a* indicates the sonic point. Substitution into the specific entropy relation (A2) then gives [69]:

(A8) $$\begin{array}{c} \frac{s\left(x\right)-s_{a}}{c_{p}}=\frac{\left(1+\gamma \right)}{2\gamma }\ln\left(\frac{1+\gamma }{\left(\gamma -1\right)\hat{M}{\left(x\right)}^{2}+2}\right)+\frac{\left(\gamma -1\right)\ln(\hat{M}\left(x\right))}{\gamma } \end{array}$$

Hence, by differentiation:

(A9) $$\begin{array}{cc} \frac{1}{c_{p}}\;\frac{ds(x)}{dx} & =-\frac{2\left(\gamma -1\right)(\hat{M}{\left(x\right)}^{2}-1)}{\gamma \left[\left(\gamma -1\right)\hat{M}{\left(x\right)}^{2}+2\right]\hat{M}\left(x\right)}\;\frac{d\hat{M}\left(x\right)}{dx} \end{array}$$

This gives the middle parts of (26) and the definition of Θ(x). Substitution for $d\hat{M}/dx$ (A3) gives the last part of (26). Finally, the local entropy production (4) for one-dimensional steady-state flow with no heat flux is $\hat{\dot{\sigma }}=d\left(\rho su\right)/dx$, which by (A4) reduces to $\hat{\dot{\sigma }}=\rho u\;ds/dx$. Substituting (A9) and dimensional scaling then gives the first parts of (26).

For the flow of dry air with γ=1.4 in a pipe with f=0.02 and $d_{H}=0.1$ m, plots of the frictional relations as functions of $\hat{M}\left(x\right)$ are shown in Figure A2. These include plots of $d\hat{M}/dx$ (A3) and Θ (26) in Figure A2a, and the fluid property ratios (A7) and (A8) and dimensionless entropy production (26) in Figure A2b. These plots confirm, for this example, the conclusions made for subsonic and supersonic flow in Section 4.1.3.

## Appendix C. Hydraulic Jumps in Open Channel Flow

By the conservation of fluid mass and momentum in a rectangular channel, the loss of energy per unit weight [J N${}^{-1}$ = m] across a normal hydraulic jump is [11,19,98,104,105]:

(A10) $$\begin{array}{c} \Delta E=\frac{{\left(y_{2}-y_{1}\right)}^{3}}{4y_{1}y_{2}} \end{array}$$

Substitution in the entropy production ${\dot{\sigma }}_{\mathrm{jump}}=\rho gQ\Delta E/T$ gives the first part of (39). The momentum equation also gives the following relations for the water depths:

(A11) $$\begin{array}{c} \frac{y_{2}}{y_{1}}=\frac{1}{2}\left(\sqrt{1+8Fr_{y_{1}}^{2}}-1\right)\;\mathrm{and}\;\frac{y_{1}}{y_{2}}=\frac{1}{2}\left(\sqrt{1+8Fr_{y_{2}}^{2}}-1\right) \end{array}$$

These are amalgamated in the second part of (39).

Substitution of (A11) into the dimensionless entropy production (40) yields, respectively:

(A12) $$\begin{array}{cc} \Pi_{\mathrm{jump}} & =\frac{gy_{1}{\left(\sqrt{1+8{\mathrm{Fr}}_{1}}-3\right)}^{3}}{16c_{p}T\left(\sqrt{1+8{\mathrm{Fr}}_{1}}-1\right)}=-\frac{gy_{2}{\left(\sqrt{1+8{\mathrm{Fr}}_{2}}-3\right)}^{3}}{16c_{p}T\left(\sqrt{1+8{\mathrm{Fr}}_{2}}-1\right)} \end{array}$$

The denominators of each expression in (A12) are positive for $Fr_{1},Fr_{2}>0$, so $\Pi_{\mathrm{jump}}>0$ or ${\dot{\sigma }}_{\mathrm{jump}}>0$ requires, respectively, $\sqrt{1+8Fr_{1}}-3>0$, hence $Fr_{1}>1$ and $\sqrt{1+8Fr_{2}}-3<0$, hence $Fr_{2}<1$. In consequence, from the second law of thermodynamics, a normal hydraulic jump can only form in the transition from supercritical to subcritical flow. This requirement will change for different assumptions, e.g., open channel transitions involving a change in flow rate or appreciable friction, or for an oblique hydraulic jump (Section 4.5.2).

## Appendix D. Frictional Gradually-Varied Open Channel Flow

For frictional gradually-varied one-dimensional flow in a rectangular open channel at steady state, the total energy of the fluid per unit weight [J N${}^{-1}$ = m] is given by:

(A13) $$\begin{array}{c} H\left(x\right)=z\left(x\right)+y\left(x\right)+\frac{U{\left(x\right)}^{2}}{2g} \end{array}$$

where *z* is the bed elevation [m], *y* is the water surface elevation [m], *U* is the cross-sectional average velocity [m s${}^{-1}$], *g* is the acceleration due to gravity [m s${}^{-2}$] and *x* is the flow coordinate [m]. Substituting q=U(x)y(x) and differentiation gives:

(A14) $$\begin{array}{c} \frac{dH(x)}{dx}=\frac{dz(x)}{dx}+\frac{dy(x)}{dx}-\frac{q^{2}}{gy{\left(x\right)}^{3}}\frac{dy(x)}{dx} \end{array}$$

Recognizing the energy slope $S=dH_{L}/dx=-dH/dx$, bed slope $S_{0}=-dz/dx$ and Froude number $Fr_{y}=q/\sqrt{g}\;y^{3/2}$ in (A14) gives:

(A15) $$\begin{array}{c} S\left(x\right)=S_{0}\left(x\right)+\left(Fr_{y}{\left(x\right)}^{2}-1\right)\frac{dy(x)}{dx} \end{array}$$

Substitution of (A15) and Q=qB into the entropy production per unit length ${\tilde{\dot{\sigma }}}_{\mathrm{open}}=\rho gSQ/T$ (41) and rescaling by $\rho c_{p}q$ gives the first two lines of (42). Differentiation of $Fr_{y}$ and substitution then yields the second last term in (42). Finally, substituting Q=qB, A=By and $P_{w}=B+2y$ into (41) gives the last part of (42).

For the open channel flow of water with q=10 m${}^{2}$ s${}^{-1}$, B=5 m, n=0.02, $c_{p}=4182$ J K${}^{-1}$ kg${}^{-1}$ and T=298.15 K [11,12], plots of the normalized water depth y/B and entropy production ${\tilde{\Pi }}_{\mathrm{open}}$ as functions of $Fr_{y}$ are illustrated in Figure A3. These are quite similar to the plots of normalized pressure and entropy production with respect to $\hat{M}$ for compressible flow in Figure A2b, and confirm, for this example, the conclusions made for subcritical and supercritical flow in Section 4.5.3.

## Appendix E. Multidimensional Phase Celerity

The wave literature provides two competing definitions of the phase celerity for multidimensional waves. These can be resolved by considering the vector wavelength λ [m], a displacement with a magnitude and direction, as illustrated for two- and three-dimensional waves in Figure A4. Each figure represents a single wavelength and its resolution into components, hence ${\left||\lambda |\right|}^{2}=\sum_{i=1}^{n}\lambda_{i}^{2}$, where *n* is the dimension. The travel time is identical for the overall wave and each component, given by the period τ [s]. The velocity (phase celerity) of the wave relative to the medium is therefore defined by:

(A16) $$\begin{array}{c} c=\frac{\lambda }{\tau },\;\mathrm{hence}\;c_{i}=\frac{\lambda_{i}}{\tau },\;\forall i\in \left\{x,z\right\}\;\mathrm{or}\;\left\{x,y,z\right\}, \end{array}$$

This gives ${\left||c|\right|}^{2}=\sum_{i=1}^{n}c_{i}^{2}$. In consequence, the vectors c and λ are parallel, generating similar triangles or tetrahedra of their components (varying only in size).

Now consider the definition of the phase celerity given in (48) (e.g., [101,117,119]):

(A17) $$\begin{array}{c} c=\frac{\omega k}{{\left||k|\right|}^{2}},\;\mathrm{hence}\;c_{i}=\frac{\omega k_{i}}{{\left||k|\right|}^{2}},\;\forall i\in \left\{x,z\right\}\;\mathrm{or}\;\left\{x,y,z\right\} \end{array}$$

where k is the vector wavenumber [m${}^{-1}$]. By definition, the angular frequency is ω=2π/τ for ω>0 and ω=−2π/τ for ω<0, independent of direction, so by substitution into and rearrangement of (A16) and (A17):

(A18) $$\begin{array}{c} \lambda =\pm \frac{2\pi k}{{\left||k|\right|}^{2}},\;\mathrm{hence}\;\lambda_{i}=\pm \frac{2\pi k_{i}}{{\left||k|\right|}^{2}},\;\forall i\in \left\{x,z\right\}\;\mathrm{or}\;\left\{x,y,z\right\} \end{array}$$

Accordingly, the vector diagrams for λ and k are also similar, as shown in Figure A4, with the possibility of λ being parallel and/or antiparallel to k, depending on the system. Equations (A16)–(A18) establish c, λ and k as vectors acting in the same direction (and/or with k in the opposite direction), each satisfying the Euclidean definition of vector magnitudes. Taking norms gives the relations:

(A19) $$\begin{array}{c} \left||c|\right|=\frac{\left||\lambda |\right|}{\tau }=\frac{\omega }{\left||k|\right|},\;||\lambda ||=\frac{2\pi }{\left||k|\right|} \end{array}$$

For one-dimensional waves with ω>0, these reduce to the known relations c=λ/τ=ω/k and λ=2π/k.

In the wave literature, several authors (e.g., [101,114,117,119]) consider the phase speed c=ω/||k||, necessarily defined in the direction of wave propagation. When multiplied by the unit normal $\hat{c}=\hat{k}=k/\left||k|\right|$, this gives the vector phase celerity (A17), so is defined correctly. However, a number of authors (e.g., [113,114,117]) then define each component of the wavelength ${\overset{˘}{\lambda }}_{i}$ and phase celerity ${\overset{˘}{c}}_{i}={\overset{˘}{\lambda }}_{i}/\tau$ by the chord between wave crests in that coordinate direction, rather than as the projection of the vector onto each coordinate axis. The relationship between these chords and the vector components are illustrated for two- and three-dimensional waves in Figure A5. By trigonometry, consideration of the triangles formed by the chord and vector components (in three-dimensional systems, by each triangular face of the tetrahedron) gives the relations:

(A20) $$\begin{array}{c} {\overset{˘}{c}}_{i}=\frac{{\left||c|\right|}^{2}}{c_{i}},\;\forall i\in \left\{x,z\right\}\;\mathrm{or}\;\left\{x,y,z\right\} \end{array}$$

As evident, the chord terms ${\overset{˘}{c}}_{i}$ in (A20) are inconsistent with components of the vector c, instead giving ${\left||c|\right|}^{-2}=\sum_{i=1}^{n}{\overset{˘}{c}}_{i}^{-2}$, with an analogous relation ${\left||\lambda |\right|}^{-2}=\sum_{i=1}^{n}{\overset{˘}{\lambda }}_{i}^{-2}$ applicable to the wavelength chords [114,117]. Synthesis with (48) or (A17) then gives [101,112,114,115,117]:

(A21) $$\begin{array}{c} {\overset{˘}{c}}_{i}=\frac{\omega }{k_{i}},\;\forall i\in \left\{x,z\right\}\;\mathrm{or}\;\left\{x,y,z\right\} \end{array}$$

In this study, we require correctly defined vector phase and group celerities for the vector Froude numbers (50) and (51), (55) and (56) and (60) and (61), for the identification of vectorial flow regimes and the calculation of angles between celerities or Froude numbers. For these reasons, this study ignores the chord definitions in (A20) and (A21), and exclusively uses the vector definition of phase celerity given in (48) or (A16) and (A17).

## Appendix F. Multidimensional Wave Dispersion

For one-dimensional waves, the phase celerity is c=ω/k; hence, the group celerity is (see (22), (35) or (43)), e.g., [63,64,99,101]:

(A22) $$\begin{array}{cc} c^{\mathrm{group}} & =\frac{d\omega }{dk}=\frac{d(ck)}{dk}=c+k\frac{dc}{dk} \end{array}$$

The derivative dc/dk thus provides a discriminator between normal (dc/dk<0, hence $c^{\mathrm{group}}<c$) and anomalous (dc/dk>0, hence $c^{\mathrm{group}}>c$) dispersion. It is necessary to find an equivalent relation between the phase and group celerities for two- or three-dimensional waves, such as internal gravity waves (Section 4.7) and inertial waves (Section 4.8).

The multidimensional phase and group celerities are in general given by (48) (e.g., [101,117,119]):

(A23) $$\begin{array}{c} c=\frac{\omega k}{{\left||k|\right|}^{2}},\;c^{\mathrm{group}}=\nabla_{k}\omega \end{array}$$

The component-wise solution of the first relation gives $\omega =c_{i}{\left||k|\right|}^{2}/k_{i}$ for i∈{x,z} or {x,y,z}. Substitution into the second and rearrangement gives the difference relation:

(A24) $$\begin{array}{c} c_{i}^{\mathrm{group}}-c_{i}=c_{i}\left(1-\frac{{\left||k|\right|}^{2}}{k_{i}^{2}}\right)+\frac{{\left||k|\right|}^{2}}{k_{i}}\frac{\partial c_{i}}{\partial k_{i}} \end{array}$$

The sign of (A24) indicates the occurrence of normal (<0), anomalous (>0) or non-dispersive waves (=0) in the *i*th direction. For one-dimensional waves, $k_{i}=k$, ${\left||k|\right|}^{2}=k^{2}$ and (A24) reduces to (A22), which includes the relations in (22), (35) or (43).
